# Soft culture substrates favor stem-like cellular phenotype and facilitate reprogramming of human mesenchymal stem/stromal cells (hMSCs) through mechanotransduction

**DOI:** 10.1038/s41598-019-45352-3

**Published:** 2019-06-24

**Authors:** Heloísa Gerardo, Ana Lima, João Carvalho, João R. D. Ramos, Sofia Couceiro, Rui D. M. Travasso, Ricardo Pires das Neves, Mário Grãos

**Affiliations:** 1 CNC — Center for Neuroscience and Cell Biology, University of Coimbra, UC-Biotech Building, Biocant Park, Cantanhede, Portugal; 2 Faculty of Science and Technology, University Nova of Lisbon (MIT-Portugal PhD Program), Caparica, Portugal; 3 Centro de Física da Universidade de Coimbra (CFisUC), Department of Physics, University of Coimbra, Coimbra, Portugal; 4 Max Planck Institute for Dynamics and Self-Organization, Göttingen, Germany; 5 Stemlab S.A. (Crioestaminal), Biocant Park, Cantanhede, Portugal; 6 Institute for Interdisciplinary Research, University of Coimbra, Coimbra, Portugal; 7 Biocant, Technology Transfer Association, Cantanhede, Portugal

**Keywords:** Mechanotransduction, Nucleus, Mesenchymal stem cells, Induced pluripotent stem cells, Reprogramming

## Abstract

Biophysical cues influence many aspects of cell behavior. Stiffness of the extracellular matrix is probed by cells and transduced into biochemical signals through mechanotransduction protein networks, strongly influencing stem cell behavior. Cellular stemness is intimately related with mechanical properties of the cell, like intracellular contractility and stiffness, which in turn are influenced by the microenvironment. Pluripotency is associated with soft and low-contractility cells. Hence, we postulated that soft cell culture substrates, presumably inducing low cellular contractility and stiffness, increase the reprogramming efficiency of mesenchymal stem/stromal cells (MSCs) into induced pluripotent stem cells (iPSCs). We demonstrate that soft substrates (1.5 or 15 kPa polydimethylsiloxane – PDMS) caused modulation of several cellular features of MSCs into a phenotype closer to pluripotent stem cells (PSCs). MSCs cultured on soft substrates presented more relaxed nuclei, lower maturation of focal adhesions and F-actin assembling, more euchromatic and less heterochromatic nuclear DNA regions, and increased expression of pluripotency-related genes. These changes correlate with the reprogramming of MSCs, with a positive impact on the kinetics, robustness of colony formation and reprogramming efficiency. Additionally, substrate stiffness influences several phenotypic features of iPS cells and colonies, and data indicates that soft substrates favor full iPSC reprogramming.

## Introduction

Cellular microenvironment biophysical cues strongly influence stem/progenitor cell behavior by regulating processes like migration^1–3^, proliferation^4–6^, differentiation^7–10^ and maintenance of pluri/multipotency^11–14^. Mammalian cells generate, sense and respond to mechanical forces transmitted from focal adhesions (FAs) through the cytoskeleton^15^ to the nucleus^16–18^. The FAs are crucial structures for cellular mechanotransduction, also providing anchorage points for attachment to the extracellular matrix (ECM). The stiffness varies within and between tissues, resulting in diverse mechanical signals sensed by cells, and represents an important component of the stem cell niche^19,20^.

Several studies correlate stemness with cellular stiffness. Nuclear rigidity increases with the differentiation state of the cell, therefore pluripotent stem cells (PSCs), multipotent cells and fully differentiated cells present increasingly stiffer nuclei^17,21^. Moreover, the overall stiffness of differentiated or multipotent cells is higher than pluripotent embryonic stem cells (ESC), but after undergoing induced reprogramming, the stiffness of the initially more committed cells decreases to levels characteristic of ESCs^22^.

Different cell types present distinctive chromatin patterns. The nuclei of pluripotent cells present more euchromatic regions than progenitor^23^ and fully differentiated^24^ cells. This seems intimately related to mechanical signals and intracellular stiffness and contractility, since the nucleus is mechanically coupled to cytoskeletal elements by the LINC (Linker of Nucleoskeleton to Cytoskeleton) complex^25^. Hence, the cytoskeleton transmits mechanical cues from the ECM to the nucleus, modulating nuclear shape, size and mechanical strain, and influencing the genomic structure^17,26–28^.

There is a strong correlation between cellular stemness, intracellular contractility and stiffness. The pluripotent state is associated with intracellular and nuclear softness and an overall relaxed state of the cytoskeleton and the cell in general. Since intracellular contractility of mesenchymal stem/stromal cells (MSCs) scales with substrate stiffness^29^, we postulated that by using soft cell culture substrates, low intracellular contractility and stiffness should be consequently achieved and reprogramming efficiency into iPSCs should increase.

The motivation to use MSCs as a target cell type for reprogramming into iPSCs is several fold. In particular, human umbilical cord MSCs (hUC-MSCs) are considered very promising in clinical settings (with several ongoing clinical trials^30^) due to advantages related with their origin (being collected at birth from extraembryonic tissues can be easily obtained and present few ethical concerns, being also less exposed to sources of infection and mutagenic agents), and for presenting a more immature state when comparing with differentiated adult cell types or MSCs obtained from other sources such as bone marrow or adipose tissue. Moreover, hUC-MSCs proliferate rapidly and are easy to maintain *in vitro*, being readily available from both public and private cryopreservation banks^31–33^. Additionally, MSCs are known to be a very mechanosensitive cell type, often used as a model in mechano- modulation experiments by several independent groups^11,29,34^. Hence, due to the fact that hUC-MSCs combine all the aforementioned advantages (clinically relevant, easy to obtain and maintain, and highly mechanosensitive), this was the cell type selected for the current study.

## Results and Discussion

### Substrate rigidity modulates nuclear shape, FAs area and actin cytoskeleton

**Figure 1:**
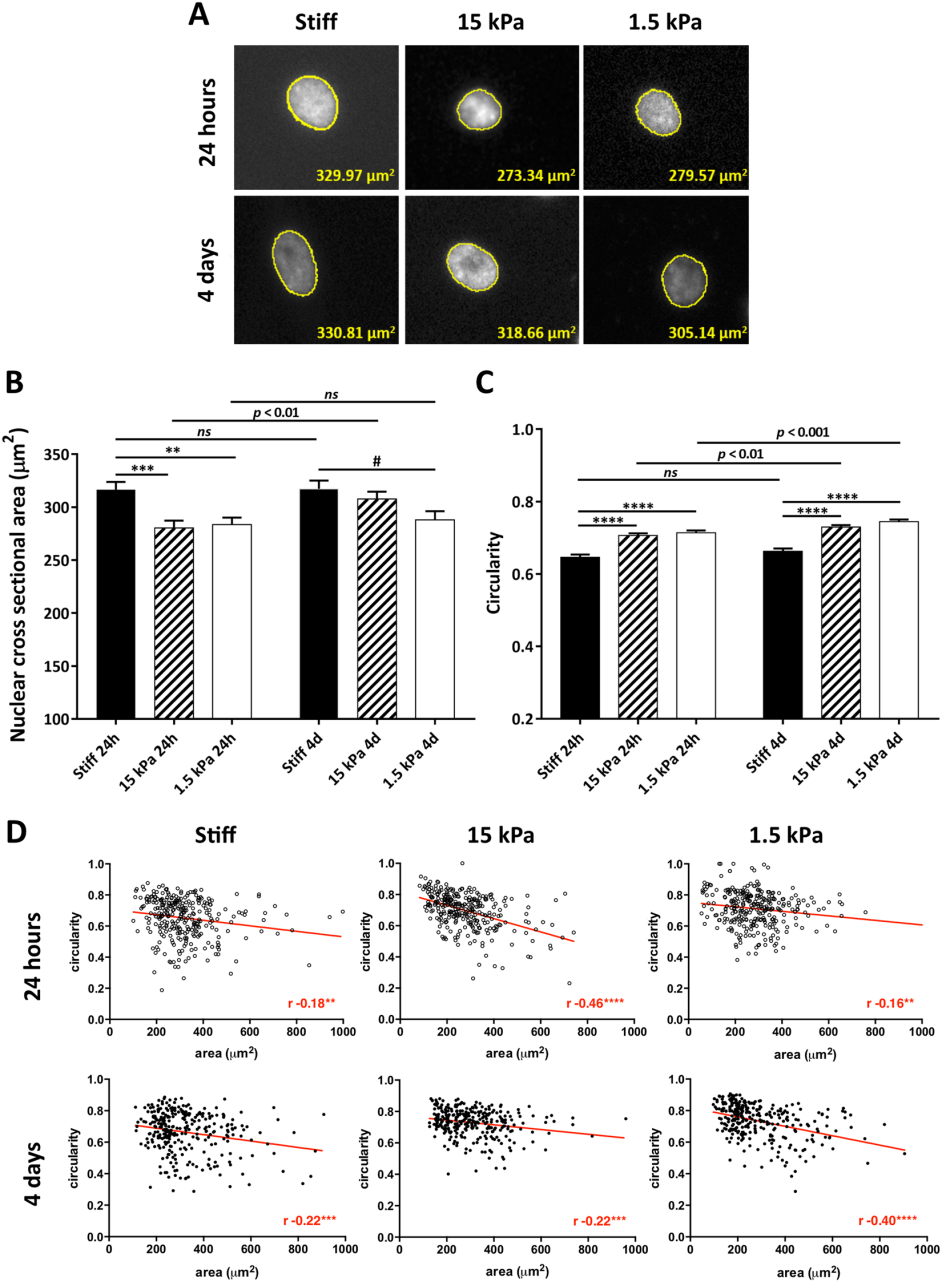
Modulation of nuclei from MSCs cultured on substrates with distinct stiffness. MSCs were cultured for the indicated time on stiff (TCPs or glass) or soft substrates (15 or 1.5 kPa PDMS). (**A**) Representative fluorescence microscopy images of nuclei of DAPI-stained MSCs cultured on TCPs or PDMS, (**B**) respective quantification of nuclear cross section, and (**C**) respective nuclear circularity measurement (mean ± SEM of 100 random nuclei per independent experiment; n = 3). Statistical analysis was performed using unpaired t-test when comparing between cells on similar substrates 24 h Vs 4d in culture (*ns*: non-significant; *p* < 0.01; *p* < 0.001) or using one-way ANOVA followed by Dunnett’s multiple comparison test when comparing between cells on distinct substrates after 24 h or 4 days (*p < 0.05, **p < 0.01, ***p < 0.001, ****p < 0.0001), using TCPs as the control. (**D**) Scatter plot of nuclear circularity and area (same data from **B** and **C**) with Pearson’s correlation analysis (**p < 0.01, ***p < 0.001, ****p < 0.0001).

Undifferentiated cells and respective nuclei present lower elastic moduli^21,22^ and lower prestress^17^ than differentiated cells. Since mechanical cues from the extracellular environment can modulate nuclear shape and tension through the actomyosin network^26^, we sought to evaluate the nuclei of MSCs maintained on substrates with distinct stiffness—GPa range (stiff glass/tissue culture polystyrene—TCPs), 15 or 1.5 kPa (soft polydimethylsiloxane—PDMS). The nuclear cross-sectional area of cells cultured on soft substrates is significantly lower than on stiff substrates in cells cultured for either 24 hours or 4 days, although for the latter time point the difference was only statistically significant between cells cultured on 1.5 kPa and stiff substrates (Fig. 1A,B). Additionally, the nuclei of cells maintained on stiff substrates present lower circularity than those on soft (Fig. 1C), and an inverse correlation could be found between nuclear cross-sectional area and circularity (Fig. 1D). Taken together, data indicates that the nuclei of cells cultured on stiff substrates suffer higher mechanical strain than on soft, in accordance with what has been described to occur in response to higher mechanical stress transmitted from the actomyosin cytoskeleton to the nucleus through the LINC complex in response to increased substrate stiffness^26^. It was reported that LINC-independent mechanisms may also contribute to nuclear flattening occurring in cells on stiff substrates, namely the expansive/compressive stresses produced by the cell membrane during cell spreading^35^.

**Figure 2:**
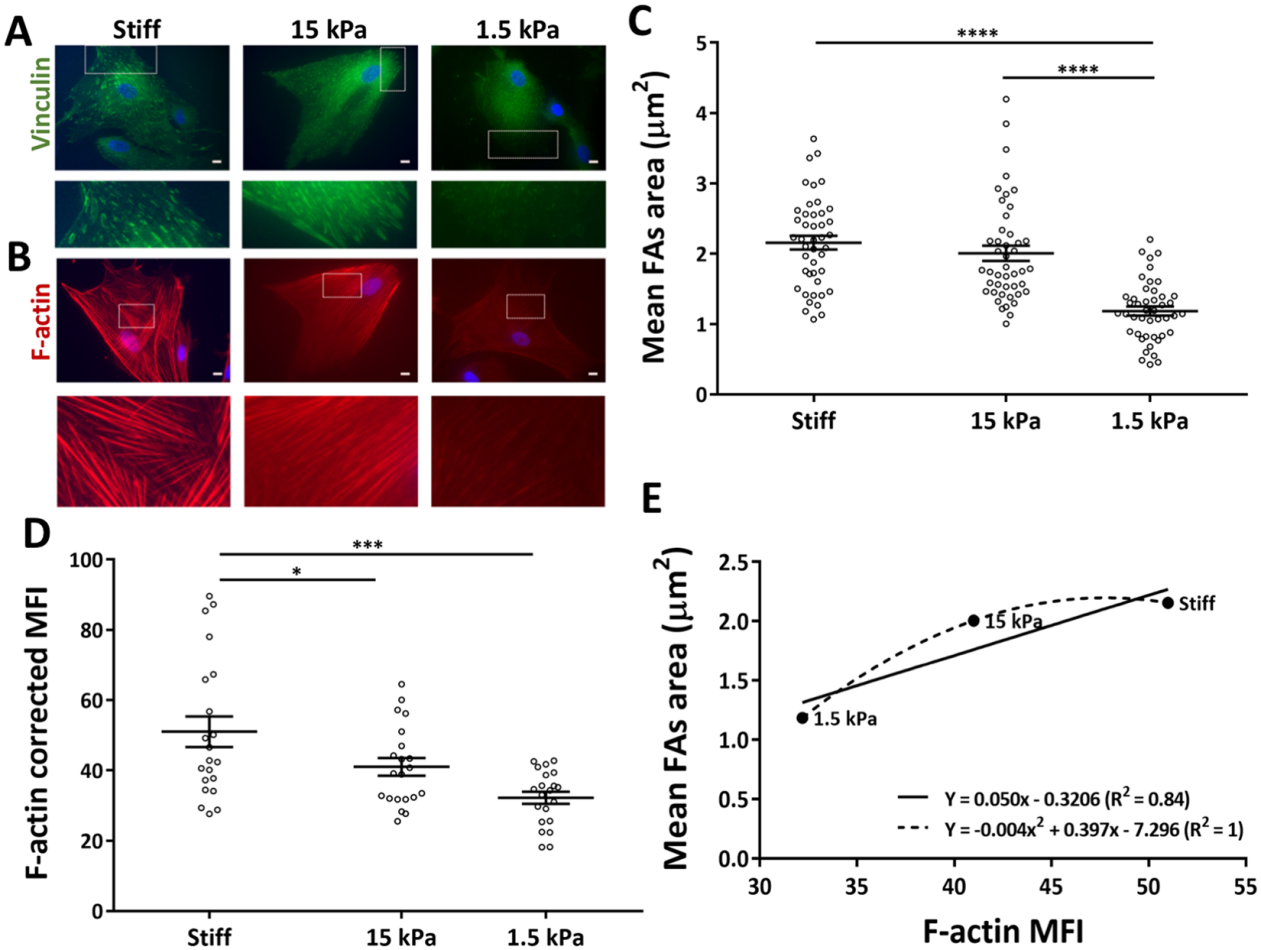
Substrates with distinct stiffness induced modulation on MSCs actin cytoskeleton and focal adhesions. Representative fluorescence microscopy images of MSCs (**A**,**B** —upper panels) cultured for 24 h on glass coverslips or PDMS, stained with (**A**) anti-vinculin antibody to assess focal adhesions or (**B**) TRITC-labeled phalloidin to assess actin stress-fibers (scale bars represent 10 μm and highlighted areas are magnified in the lower panels). Highlighted areas in (**A**) are representative of the lamellar zones selected for data analysis in (**C**), representing the average FA area per cell (dots) and mean ± SEM of the mean FA area of at least 14 cells per independent experiment (n = 3). Statistical analysis was performed using one-way ANOVA followed by Tukey’s multiple comparison test (****p < 0.0001). F-actin content (**B**) was assessed by MFI quantification (**D**) of at least 7 cells per independent experiment (n = 3). Statistical analysis was performed using one-way ANOVA followed by Dunnett’s multiple comparison test (*p < 0.05, ***p < 0.01). (**E**) The linear and quadratic regressions indicate the increase of focal adhesions area with actin stress-fibers content.

FAs and actin stress fibers are major elements of cellular mechano-sensing and effector machinery^15–17^. It is known that in MSCs larger FAs occur when higher traction forces are exerted by the cell on a given substrate, and that FAs reinforcement is dependent on actomyosin tension^11,29^. Thus, to gain some insight into the level of intracellular contractility and the traction force exerted by MSCs, the areas of FAs and F-actin content were analyzed on cells cultured on stiff or soft substrates (Fig. 2). The mean FAs area was measured within the lamellar zone of the cell edge^36,37^ (Fig. 2A), and it is significantly lower in cells cultured on 1.5 kPa, when compared with those cultured on 15 kPa or stiff substrates (Fig. 2C). These results suggest that substrate stiffness influences FAs area in a direct manner, as described previously^11,29,36^.

Cells cultured on 1.5 kPa, 15 kPa or stiff substrates presented increasingly well-assembled and organized F-actin cytoskeleton (Fig. 2B), indicating that cells cultured on the latter substrates were under higher actin-mediated tension. Quantification of F-actin present on MSCs cultured on soft substrates (1.5 or 15 kPa) confirmed a significant decrease comparing with cells on stiff substrates (Fig. 2D). Overall, it could be observed that cells cultured on increasingly stiffer substrates present larger focal adhesions and more robust actin stress fibers (Fig. 2E), as expected^11,29^.

Taken together, these results suggest that MSCs cultured on soft substrates exert lower traction force on their respective substrate, as indicated by the simultaneous presence of less robust FAs (Fig. 2A,C), lower F-actin content (Fig. 2B,D), and apparently more relaxed nuclei (Fig. 1). The positive correlation between FAs size at the leading edge and increased cell traction force seems to be positive for MSCs and fibroblasts^11,29,36^, although for the latter, it was reported that such correlation only exists for FAs larger than 1 µm^2,38^. Although we could not find an equivalent observation in the literature for MSCs, in our study the average FA size is larger than the referred area. Another exception reported in the literature is that a negative correlation seems to occur between FAs size and traction force specifically at the leading edge of actively migrating cells (in a model of goldfish fin fibroblasts)^39^.

### MSCs undergo chromatin remodeling and enhanced expression of endogenous pluripotency- related genes in response to soft substrates

**Figure 3:**
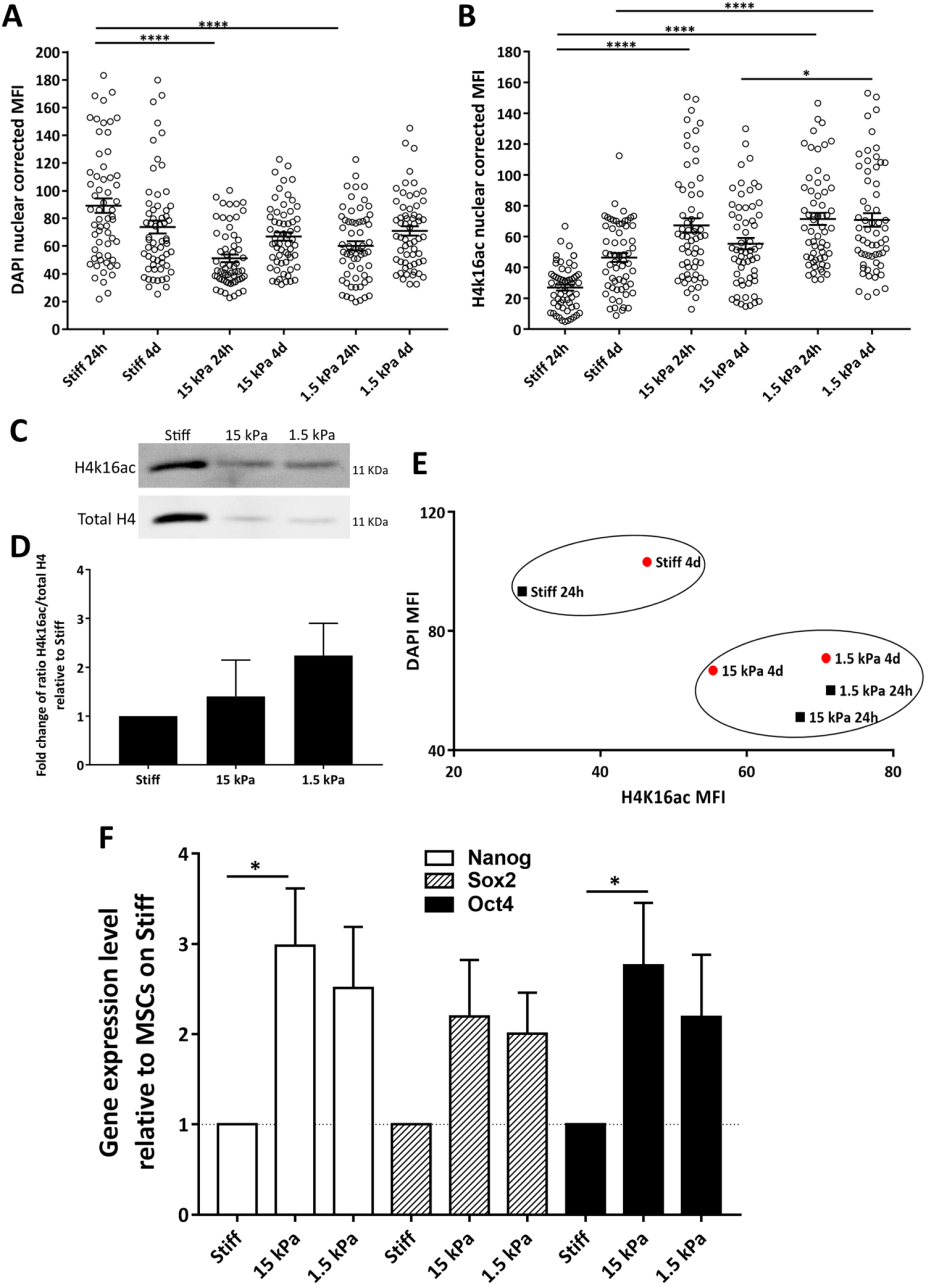
Soft substrates favor euchromatin and expression of endogenous pluripotency-related genes by MSCs. To assess (**A**) chromatin condensation and (**B**) euchromatin content, mean fluorescence intensity values of DAPI and H4K16ac stained nuclei (respectively) were quantified (20 nuclei per independent experiment) and represented as mean ± SEM (n = 3) in MSCs after 24 hours or 4 days in culture. Stiff substrates correspond to TCPs (**A**) or glass coverslips (**B**). (**A**,**B**) Statistical analysis was performed using one-way ANOVA followed by Tukey’s multiple comparison test when comparing cells on distinct substrates after 24 h or 4d in culture (*p < 0.01, ****p < 0.0001). (**C**) Representative western blot analysis of nuclear extracts obtained from MSCs cultured for 24 hours on TCPs (stiff) or PDMS (1.5 kPa, 15 kPa) probed with antibodies specific for H4K16ac and total H4, and respective quantification in (**D**) as fold change of H4K16ac/total H4 ratio (relative to the stiff substrate). Data represent mean ± SEM (n = 4). **(E)** Scatter plot relating DAPI and H4K16ac MFI (data from **A** and **B**). (**F**) Gene expression analysis of pluripotency-related genes (Nanog, Oct4 and Sox2) expressed by MSCs cultured for 4 days on distinct substrates was assessed by qRT-PCR. Bars represent mean ± SEM of at least 3 independent experiments and are expressed as fold change of 2^−ΔΔCt^ using TCPs (stiff) as the control condition. Statistical analysis was performed using Kruskal-Wallis test followed by Dunn’s multiple comparison test (*p < 0.05) in (**D**,**F**).

Pluripotent stem cells present a dominance of euchromatin, gaining more heterochromatic regions during differentiation^23,24^. Additionally, mechanical stimuli can influence chromatin organization and gene expression in distinct cell types^27,28,40–42^. Thus, we analyzed the influence of substrate stiffness on the chromatin state and expression of pluripotency-related genes in MSCs (Fig. 3).

The heterochromatic regions, which can be detected as bright fluorescence spots in DAPI-stained nuclei corresponding to higher mean fluorescence intensity (MFI) regions, have high levels of chromatin compaction^23^, whereas regions rich in histone H4 acetylated on lysine 16 (H4K16ac) are euchromatic^43^. Quantitative analysis of fluorescence microscopy images of MSCs plated for 24 hours on soft substrates presented significantly lower DAPI MFI, (Fig. 3A and Supplementary Fig. S1) and higher H4K16ac MFI (Fig. 3B and Supplementary Fig. S1) than those cultured on stiff substrate, hence indicating lower heterochromatic and higher euchromatic content in cells on the former substrates. Western-blot analysis of euchromatic content revealed the same tendency (Fig. 3C,D). Significantly higher euchromatic content was also observed when comparing MSCs cultured for 4 days on 1.5 kPa substrates with those on 15 kPa or stiff matrices (Fig. 3B). By plotting the data reflecting the MFI of DAPI Vs H4K16ac acquired from MSCs cultured for 24 h or 4d on substrates with distinct stiffness, there was a clear tendency for clustering all conditions into two groups, one constituted by the stiff and the other by the soft (1.5 and 15 kPa) substrate conditions (Fig. 3E). This further illustrates that MSCs maintained on a high stiffness substrate tend to have high heterochromatic content (high DAPI MFI) and low levels of euchromatin (low H4K16ac MFI) and the opposite for those on soft substrates (at least during the tested timepoints of 24 h and 4 days). These results are similar to what is observed when chromatin is relaxed by hypoosmotic stimuli (increase in H4K16ac) or condensed by hyperosmotic conditions (decrease in H4K16ac). Together these results seem to indicate that the chromatin mark H4K16ac, a good sensor for gene expression and chromatin accessibility, is changing quickly in response to biophysical cues^43,44^.

Next, we investigated whether substrate stiffness could modulate the expression of pluripotency-associated genes. Expression of Nanog, Sox2 and Oct4 by qRT-PCR analysis showed a trend of increased expression of these genes in MSCs plated on soft substrates when compared to the stiff substrate (Fig. 3F), in line with what was already described for fibroblasts^41^. Increased expression of Oct4 and Nanog was also described to occur in HEK cells cultured on soft low-adhesion substrates (compared to cells attached to stiff glass coverslips) that favor the formation of spherical cell clusters^45^, reinforcing the idea that biophysical cues and cell shape influence the expression of pluripotency-related genes in distinct cellular and microenvironment contexts.

Since the structural integrity of actin cytoskeleton is crucial for transduction of mechanical stimuli^46–48^, it seems reasonable to postulate that the less evident increased expression of Nanog, Sox2 and Oct4 (in comparison with cells on stiff substrates) observed in MSCs cultured on the 1.5 kPa substrate than on 15 kPa might be due to the lower F-actin content and organization detected on cells cultured on the former platform (Fig. 2B,D). Cells on the 1.5 kPa substrate presented poorly assembled and somehow disorganized actin cytoskeleton that may cause impaired mechanotransduction from the extracellular environment to the nucleus^46–48^.

Taken together, our data strongly suggests that by seeding MSCs on soft substrates it is possible to assign them characteristics closer to PSCs, namely more relaxed nuclei, smaller FAs, fewer stress fibers, higher euchromatic and lower heterochromatic content, and expression of pluripotency-related genes (Figs 1–3).

### Substrates with lower elastic modulus enhance cell reprogramming

Distinct mechanical stimuli seem to influence the maintenance and induction of cellular pluripotency^12,13,40^, and our data indicate that soft substrates modulate the phenotype of MSCs in a way that predictably will favor reprogramming into an iPSC state. Hence, we sought to elucidate whether substrate rigidity could modulate induced reprogramming of MSCs.

**Figure 4:**
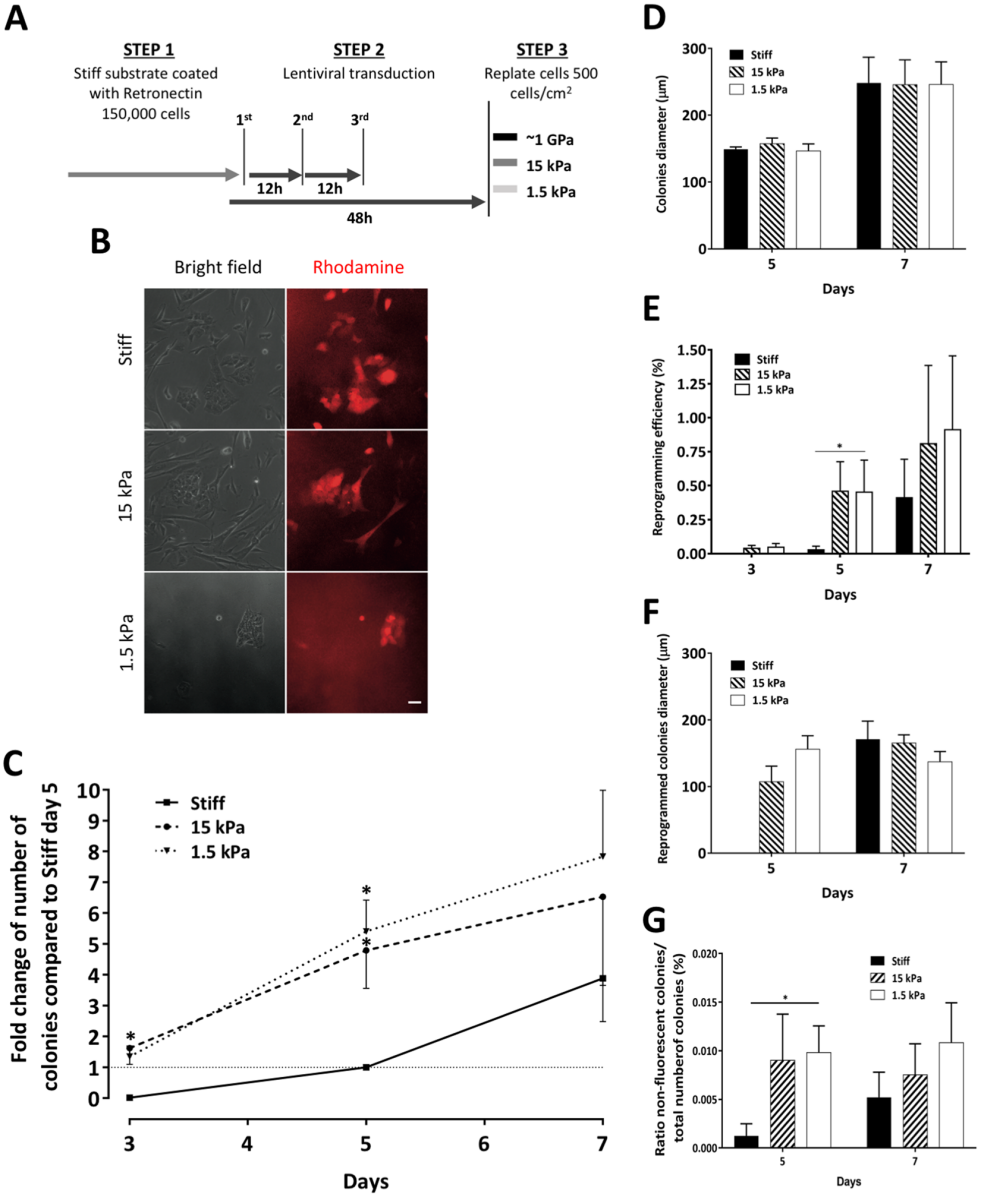
Substrates with lower elastic modulus improved kinetics and efficiency of MSCs induced reprogramming. (**A**) Schematics of the reprogramming procedure. Cells were cultured on distinct substrates for 7 days (step 3). (**B**) Representative images of newly-formed iPSCs colonies in distinct substrates (day 5), scale bar corresponds to 50 μm. (**C**) Evaluating the reprogramming kinetics, each point represents the number of newly formed colonies, at the respective time point, normalized to the number of colonies on TCPs at day 5. Points represent mean ± SEM of 3 (days 3 and 7) or 4 (day 5) independent experiments. Colonies diameter is represented in (**D**), bars represent mean ± SEM of 3 independent experiments. The reprogramming efficiency can be observed in (**E**), bars represent mean ± SEM of 3 (day 3 and 7) or 4 (day 5) independent experiments. In (**F**) is represented the diameter of reprogrammed colonies, bars represent mean ± SEM of 3 independent experiments. (**G**) Ratio of reprogrammed colonies (non-fluorescent) per total number of newly-formed colonies. Statistical analysis was performed uisng Kruskal-Wallis test followed by Dunn’s multiple comparison test (*p < 0.05) in **(C**–**G)**.

MSCs were transduced with a lentiviral reprogramming vector^49^, then seeded onto substrates with distinct rigidity (Fig. 4A) and subsequently monitored from day 3 to 7 after replating, to assess the kinetics and efficiency of the overall process (Fig. 4B–G). All the newly-formed colonies composed by iPSC-like fluorescent cells were counted, as well as those colonies whose cells ultimately lost expression of the vector-encoded fluorescent dTomato reporter, hence effectively fully reprogrammed into iPSCs^49^ (please see the Methods section and Supplementary Fig. S2).

There was a clear increase of newly-formed iPSC-like colonies (Fig. 4B) when comparing the 1.5 or 15 kPa matrices with the stiff substrate, indicating that soft substrates correlate with enhanced reprogramming of MSCs into iPS-like colony-forming cells (Fig. 4C). Significant differences were observed at days 3 and 5, but not at day 7, which may be explained at least in part by underestimating colony number at the latter day on soft substrates due to colony coalescence.

**Figure 5:**
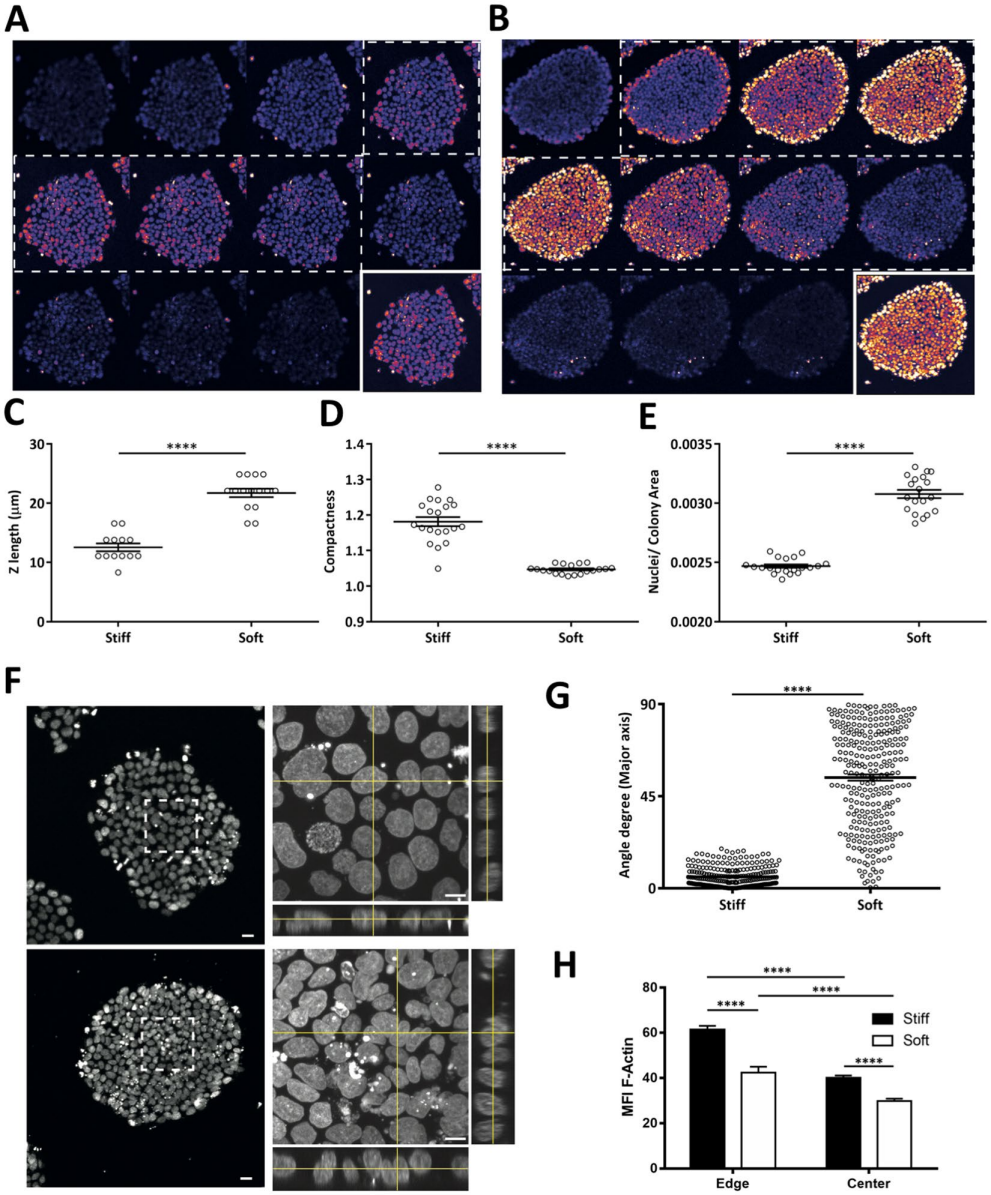
Modulation of hiPSC colonies cultured on substrates with distinct stiffness. Representative Z-stack fluorescence microscopy images of DAPI-stained hiPSCs cultured on stiff (**A**) or soft PDMS (**B**) for 4 days. (**C**) Quantification of colonies height is presented by the mean ± SEM of at least 13 colonies. The colonies compactness (i.e. the mean squared distance of the object’s pixels from the centroid divided by the area — a filled circle will have a compactness of 1, with irregular objects, or objects with holes, having a value greater than 1) is presented in (**D**) and evidenced in (**E**). These quantifications are represented as mean ± SEM of at least 19 colonies. (**F**) Representative images of nuclei morphology from colonies cultured on stiff (upper panel) and soft (lower panel) substrates. Highlighted areas are magnified in the right panels, with Z-perspective of the nuclei. Scale bars correspond to 20 μm and 10 μm in low and higher magnification images, respectively. The nuclei projection in Z, given by the major axis, was quantified and presented in (**G**) as mean ± SEM from approximately 300 nuclei. (**H**) F-actin content in the center and the edge of colonies cultured on stiff or soft substrates, assessed by MFI quantification of at least 8 colonies, bars represent mean ± SEM. Statistical analysis was performed using two tailed t-test (****p < 0.0001) in (**C**,**D**,**E**,**G**), and two-way ANOVA followed by Sidak’s multiple comparison test (****p < 0.0001) in (**H**).

Colony diameter on the distinct substrates at days 5 or 7 showed no significant differences (Fig. 4D). It is however worth noting that colony growth seems to have occurred at a faster rate on stiff substrates. Although there were practically no colonies at day 3 on the stiff substrate (Supplementary Fig. S3), the ones that formed, between days 3 and 5, acquired a similar diameter to those that formed earlier on soft substrates (Fig. 4D). This may be due to higher cell proliferation rate or larger cell diameter on the stiff substrate. Data presented in Fig. 5 seems to favor the latter hypothesis, although this issue is not under the direct scope of this study.

Reprogramming efficiency, i.e., considering only the non-fluorescent colonies — which are the ones that effectively become fully reprogrammed — divided by the initial number of transduced cells, was also favored by the soft substrates (Fig. 4E), with a significant difference found at day 5 when comparing cells on 1.5 kPa and stiff substrates. Again, the diameter of the colonies showed no significant differences between the distinct substrates (Fig. 4F).

Additionally, by calculating the frequency at which iPSC-like colonies (positive for dTomato) became fully reprogrammed (dTomato silenced), there is also a tendency for full reprogramming to occur more often on soft substrates (Fig. 4G). Although we only found statistical differences at day 5 between 1.5 kPa and stiff substrates (due to high variability in the number of colonies formed across independent experiments), the trend is present for all soft conditions (1.5 or 15 kPa) when compared with stiff.

The colonies generated were consistent with the phenotype of iPSCs, retaining a stable hESCs-like morphology after mechanical and enzymatic expansion on feeders (cultured on stiff TCPs 6-well plates) for several passages, (Supplementary Fig. S4A) and expressing pluripotency markers SSEA-4, TRA-1–60 and TRA-1–81 (Supplementary Fig. S4B), some of the most commonly used surface markers to identify human pluripotent stem cells^50,51^. In a recent study (Lima *et al*.)^44^, using the same viral vector and similar transduction protocol as described here, we have shown the reprogramming of CD34^+^ mononuclear hematopoietic stem cells and fibroblasts into iPSCs. Also, many other independent groups have successfully used the lentiviral vector described by Warlich *et al*.^49^ to reprogram distinct cell types.

Taken together, the results suggest that soft substrates allow MSCs to acquire a more relaxed state, which correlates with the enhanced formation of iPSC-like colonies (i.e., colonies of iPSC-like cells still expressing the exogenous reprogramming factors, and consequently the fluorescent marker dTomato) at earlier time-points when compared with cells on a stiff substrate (Fig. 4C). We also show that full reprogramming of iPSC-like into iPSC colonies (i.e., colonies that eventually start expressing endogenous pluripotency genes and shut down the exogenous reprogramming factors, consequently losing the fluorescent marker) correlates with soft substrates (Fig. 4G). Both effects result in enhanced reprogramming efficiency of MSCs into fully reprogrammed colonies (Fig. 4E).

**Figure 6:**
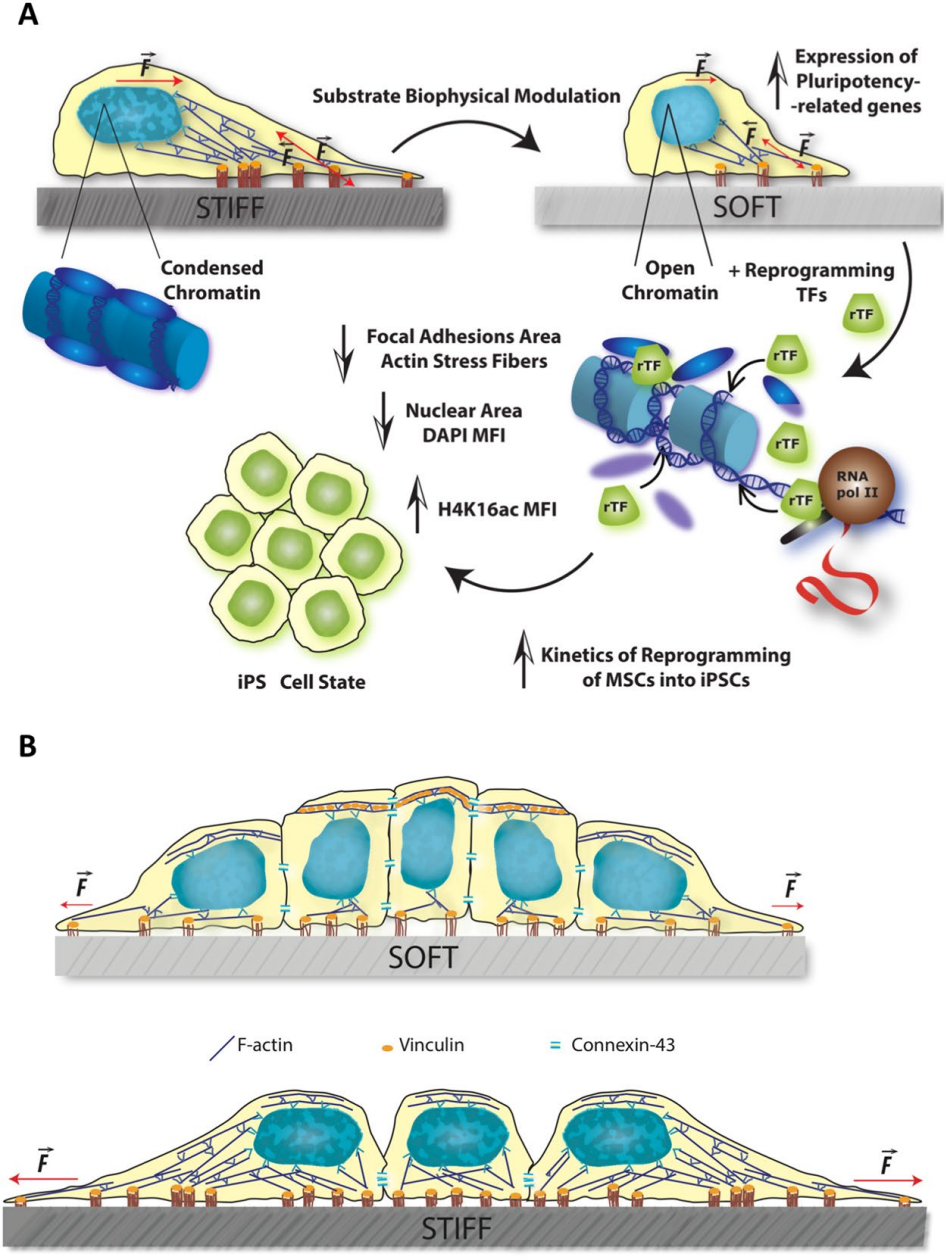
Schematics illustrating the proposed model of biophysical modulation by substrate rigidity. (**A**) Soft substrates lead to decreased focal adhesions maturation, stress fibers content and nuclear stretching in hMSCs. The subsequent increase in open chromatin nuclear regions and enhanced expression of endogenous pluripotency-related genes facilitate the induced-reprogramming of hMSCs into iPSCs by exogenous reprogramming factors. (**B**) Differences in focal adhesions maturation, stress fibers content and nuclear stretching between distinct substrates observed in iPSC colonies. Stiff substrates lead to flatter and stretched colonies with higher content of F-actin. On soft substrates, colonies are more compact, have higher projection in Z and present apical vinculin. This pattern is only excluded at the edge of the colony, where cells resemble the ones on stiff substrates.

The possible mechanisms contributing to control stiffness-modulated reprogramming efficiency are summarized in Fig. 6A. In comparison with stiff, soft substrates lead to decreased focal adhesions maturation, stress fibers content and nuclear stretching in hMSCs. We speculate that the nuclear relaxation occurring in cells cultured on soft substrates causes — or at least facilitates — changes in the chromatin epigenetic landscape, as indicated by data presented in Fig. 2A–D denoting an overall increase of euchromatin and corresponding decrease of heterochromatin. Although the mechanistic details will only be dissected in future studies, we envisage that such changes might have an important consequence during reprogramming, namely by facilitating the access of the exogenous reprogramming transcription factors (*i.e*., the Yamanaka factors) to their target genes, resulting in facilitated transcription of said targets and enhanced expression of endogenous pluripotency-related genes, facilitating the induced-reprogramming of hMSCs into iPSCs. In support of this idea, the concept of mechanoepigenetics has been recently proposed to describe mechanisms involving force-induced physical changes to chromatin, which *per se* may be responsible for some degree of direct transcriptional regulation, but that also seem to turn chromatin more prone to appropriate enzyme-mediated biochemical modifications^10,52^. It has been reported that microtopography elements (microgrooves) influence the epigenetic state of chromatin (in non-transduced cells) and consequent reprogramming efficiency of mouse or human fibroblasts into iPSCs (after transduction with the Yamanaka factors). Such mechanical cues led to increased histone H3 acetylation (AcH3) and methylation (H3K4me2 and H3K4me3) marks associated with transcriptional activation, through a mechanism that is actin cytoskeleton-dependent and involves the decrease of histone deacetylase (HDAC) activity and upregulation of WDR5 expression (a subunit of H3 methyltranferase)^40^. Conversely (although not in a context of cell reprogramming), it was recently shown that biaxial cyclic mechanical strain led to increased trimethylation of histone H3 on lysine 27 (H3K27me3, a heterochromatin mark causing persistent gene silencing) and consequent gene repression in human and mouse primary epidermal keratinocytes. The underlying mechanism involves force transmission to the nucleus by emerin (a nuclear envelope protein), actin cytoskeleton and non-muscle myosin-IIA (the NMM-II inhibitor blebbistatin prevented strain-induced epigenetic changes and gene silencing)^53^. Overall, our proposed model depicted in Fig. 6A is consistent with the literature, and new insights may be provided in future studies.

### Substrate stiffness modulates the phenotype of human iPS cells and colonies

The results in terms of kinetics and efficiency of full reprogramming suggest that besides influencing various aspects of MSCs, substrate stiffness could also affect iPSCs behavior, hence we sought to explore this idea further.

Confocal microscopy analysis of Hoechst-stained iPS cells plated on stiff (glass) or soft (1.5 kPa PDMS) substrates (Fig. 5A,B, respectively) revealed that the colonies acquired different characteristics with time. After 3–4 days in culture, colonies from both conditions were composed by a monolayer of cells but the colonies formed on the soft substrate had a more prominent 3D component (Fig. 5B), presented higher *z*-axis length (Fig. 5C), were more compact (Fig. 5D; 1 = compact, >1 less compact) and displayed higher density of nuclei per colony area (Fig. 5E), than those on the stiff matrix. Orthogonal projections revealed that the nuclear topography of iPSCs cultured on the soft substrate was distinct from those on the stiff matrix (Fig. 5F and Supplementary Video 1), except for the few outermost concentric lanes of cells (Supplementary Fig. S5), which were essentially identical to those on stiff. By measuring the angle established between the direction of the major nuclear axis with the plane of the cell culture substrate, it became evident that the nuclei of iPS cells cultured on the soft matrix present a wide dispersion of directions (mean ± SEM = 54.1° ± 1.51), several being nearly perpendicular and very few parallel to the substrate plane (Fig. 5F,G), whereas those on the stiff substrate were essentially parallel to the substrate plane (mean ± SEM = 9.3° ± 0.79), indicating high intracellular actomyosin contractility of the latter in response to elevated substrate stiffness (which is transmitted between FAs and the nucleus essentially parallel to the substrate plane^26^). These observations suggest that cells on soft substrates present a more columnar shape, in agreement with previous observations reported by culturing hESCs on soft (0.4 kPa) Vs stiff (60 kPa) Matrigel-coupled polyacrylamide gels^54^. The distribution of vinculin along the *z*-axis of the cells further supports this idea (Supplementary Video 2). iPSCs in the center of colonies cultured on the soft substrate present apical vinculin, detected as a linear staining along cell-cell contacts at a *z* consistent with the apical region of the cells (near the apex of the colony), a region also enriched in connexin-43 (Cx43), whereas at a *z*-distance close to the substrate (near the bottom of the colony) vinculin staining was diffuse and in dash-like structures, consistent with focal adhesions (Supplementary Figs. S6 and S7). In contrast, cells on the edge of iPSC colonies cultured on the soft substrate (Supplementary Fig. S7) and iPS cells on the stiff substrate (both on the edge and center) lacked organized apical vinculin and presented only diffuse distribution near the top of the colony and dash-like and diffuse distribution at a low *z*-level, typical of non-polarized cells (Supplementary Fig. S6). This bimodal distribution pattern of vinculin described here for colony-center iPSCs on the soft substrate (linear for apical vinculin and dash-like for FA-associated vinculin) is similar to what was previously reported to occur in polarized epithelial cells^55,56^. This reinforces the idea that iPS cells on the soft substrate adopt in fact a more columnar shape (epithelial-like), and seem to establish reinforced apical cell-cell contacts (highlighted by the presence of apical vinculin and Cx43), in contrast to those on the edge and/or stiff matrix. Apical-basal cellular polarization has been reported in hESCs^57^ and apical vinculin at cell-cell contacts was reported during the initial steps of development of the mouse embryo, until the morula stage^58^.

Analysis of the nuclear disposition (namely the angle with the substrate plane) and shape (Fig. 5F,G) suggested that iPSCs on the stiff substrate are experiencing higher intracellular actomyosin tension occurring between the nucleus and focal adhesions (transmitted essentially parallel to the substrate plane^26^) than those on the soft matrix, somehow resembling what was observed for MSCs on stiff Vs soft substrates (Fig. 1). This was corroborated by analyzing F-actin content. Cells on the stiff substrate displayed significantly higher F-actin content than those on the soft matrix (Fig. 5H), indicating higher actomyosin tension on the former. It could also be observed that cells on the edge of colonies on each substrate presented significantly higher F-actin content than those in the center, indicating higher intracellular tension on edge cells comparing with those in the center of the colonies (Fig. 5H, Supplementary Figs S6 and S8), in agreement with recent observations in human iPSCs^59^. It is interesting to note that spontaneous differentiation of pluripotent stem cells typically occurs at the edge of overgrown colonies^60^, which is consistent with the idea that low intracellular contractility is important for maintenance of stemness. In summary, cells on the colony edge on both stiff and soft substrates seem to be under high intracellular tension (displaying high F-actin content), present nuclei aligned with the substrate plane, FA-associated vinculin staining and lack of organized apical vinculin (Fig. 6B).

On the other hand, cells in the center of iPSC colonies cultured on a soft substrate seem to be columnar, more relaxed and present reinforced cell-cell interactions, displaying apical vinculin (Fig. 6B). The fact that cells in the center of iPSC colonies on soft substrates are the most relaxed (Fig. 5H) is in agreement with the observations in terms of nuclear topography (Fig. 5F,G and Supplementary Fig. S5).

Overall, data is compatible with the idea that iPS cells on the center of colonies cultured on the soft substrate present lower intracellular contractility and lower traction forces on the substrate (compatible with the columnar shape) than those on stiff matrices (Fig. 5B). We speculate that the relaxed state of cells on soft matrices may favor the full reprogramming of iPSC-like colonies, as observed in Fig. 4E,G. This hypothesis is in line with reports indicating that soft substrates promote the maintenance of pluripotency of mouse embryonic stem cells (mESCs)^13^ and that transmission of increasing force via integrins leads to reduced expression of pluripotency genes in the same cell type^61^. Further elucidation of this issue will be addressed in future studies.

### Mathematical model predicts that low traction force favors full reprogramming to pluripotency

A model to describe mesenchymal stem cells (MSC) genetic reprogramming to pluripotency, as measured in experimental observations, was implemented using a Cellular Potts Model (CPM), described elsewhere^3,62^. The main parameters used are presented in Supplementary Table S1.

The model is based on two major assumptions about the behavior of cells on substrates of different rigidity. The first one is that cells exert a higher traction force on a more rigid matrix, as shown by different experimental observations^13,63,64^. In fact, due to the cell’s limited energy supply, the mechanical work exerted by the cell on the substrate is approximately constant^63^. Therefore, in rigid substrates the cell applies a greater force than in softer substrates, where a smaller force is enough to achieve large displacements. At very rigid substrates the force exerted by the cell saturates when it reaches its limit in the formation of focal adhesions^64^. As mentioned above, the mechanical displacement of the Extracellular Matrix (ECM) is a source of mechanosensing signal for cell differentiation and also for cell-cell communication^3,62^. In this model, the medium rigidity is described by its Young’s modulus *E* and the cell traction force, *µ*, increases with *E* according to the expression

1 $$\mu =a+\frac{AE}{b+E}$$

where *a*, *A* and *b* are parameters adjusted to experimental data and given in Supplementary Table S2. The data used is from Sun *et al*.^64^ where the total traction forces per cell were measured, using single hESCs on PDMS micropost arrays with different rigidities.

**Figure 7:**
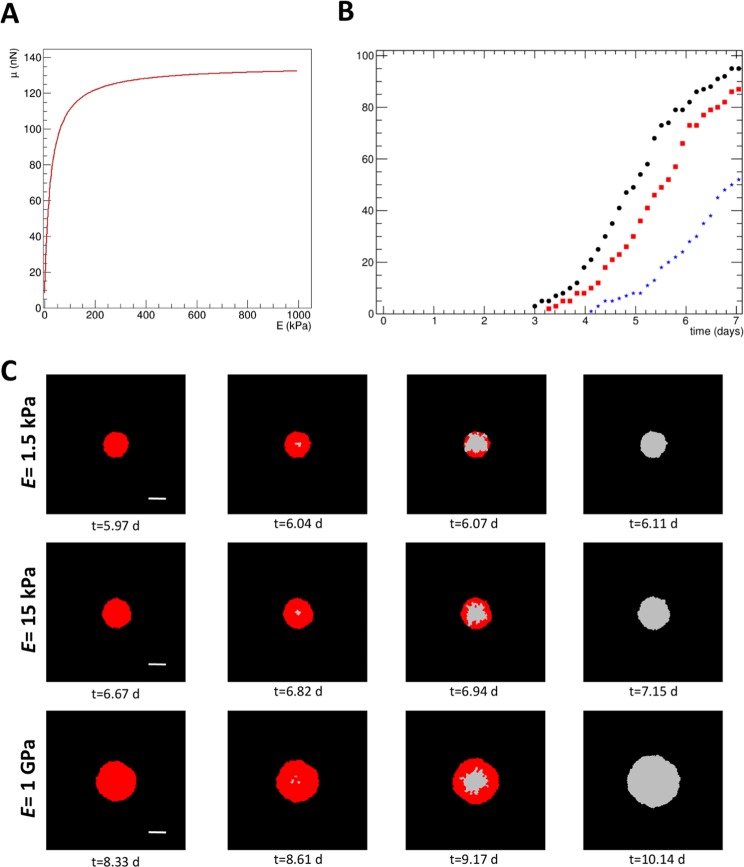
Model of MSC reprogramming to pluripotency in different rigidity substrates. (**A**) Traction force, *µ*, as a function of substrate Young’s modulus, *E*, as given by equation 1. (**B**) Cumulative distribution of colony reprogramming, for soft (black points), intermediate (red squares) and rigid (blue stars) substrates, as a function of time. (**C**) Examples of typical simulations of colonies growing in substrates with different rigidities. The reprogrammed cells are represented in grey and the non-reprogrammed in red. The scale bars (on the first column) correspond to 100 µm. The simulation indicates that the reprogramming starts close to the colony center and moves quickly towards the periphery. Typically, the cell reprogramming within the same colony started later and was slower in stiffer substrates.

From published data it is clear that the relationship between the traction force and *E* is not linear and that it saturates for high values of *E* (maximum value is *a*+*A* and the non-constant term is half-maximum for *E* = *b*). This function is shown in Fig. 7A.

For the second assumption, we have shown that MSC reprogramming to iPSCs is faster on a softer matrix. As the cell exerts a lower traction force on the substrate, it is more relaxed (Figs 1 and 2), the nucleus has a lower level of chromatin compaction (Fig. 3), and the expression of endogenous pluripotency-related genes is higher (Fig. 3F), hence facilitating reprogramming. Full reprogramming of the newly formed iPSC-like colonies also seems to be facilitated by soft substrates, presumably due to lower traction forces being exerted on the substrate, which is the assumption being tested in the current model. In the present computational model reprogramming is described by a quantity called *R*, for reprogramming level, with values between 0 (start of reprogramming) to 1 (fully reprogrammed). The rate of change of reprogramming is a function of the total traction force exerted by the cell on the matrix (i.e., of the integral of the traction *µ* on the cell surface), so that on a softer matrix there is a lower total force exerted by the cell and its reprogramming is faster. In a more rigid matrix the force is higher and the reprogramming slower. If the cell is in a confined space, like in the middle of a colony, the cell area that touches the surface is smaller, and so the force it exerts is also lower, leading to a faster reprogramming rate. For further detailed explanation about the mathematical model, please see the Methods section.

Table 1 shows some average results obtained with the simulation model after 500 Monte Carlo Steps (MCS), corresponding to about 7 days. The cumulative distribution for colony reprogramming, as a function of culture time, in the three different substrates, is presented in Fig. 7B. At day 5 on a soft matrix (*E* = 1.5 kPa) about half of the colonies that will eventually undergo full reprogramming have already reprogrammed, while for *E* = 15 kPa only about 30% have reprogrammed. At day 7 all the colonies grown on the soft matrices and about 90% on *E* = 15 kPa have already reprogrammed, while only about half of the ones on the rigid matrix (*E* = 1 GPa) have reprogrammed. These reprogramming times are in agreement with what was observed before in different cell contexts using the same reprogramming vector^44,49^.

**Table 1:** Results from the model after 500 MCS (about 7 days). The uncertainties presented are the standard deviations of 100 runs.

*E* (kPa)	Reprog. time (days)	Colony diameter (µm)	Number of cells
1.5	5.2 ± 1.4	189 ± 17	167 ± 29
15	5.7 ± 1.4	192 ± 15	172 ± 26
1 × 10^6^	6.9 ± 1.4	191 ± 17	171 ± 29

The model also suggests that the cells in a colony reprogram in an almost synchronous manner in softer substrates, with typically the cells at the center of the colony being slightly faster to reprogram than the cells at the periphery (Fig. 7C). Also, the differences in reprogramming time in the model within the same colony were typically larger in stiffer substrates. These predictions remain to be experimentally tested in future work.

The hypothesis that the low intracellular contractility observed in cells cultured on soft substrates (Figs 1, 2, 5 and 6) results in low traction force exerted on the substrate and that this favors reprogramming of MSCs into iPSCs was tested using the mathematical model. The results obtained suggest that this concept is valid and deserves further attention in future work.

## Conclusion

Our findings support the idea that stemness is intimately related with the biophysical microenvironment and mechanical properties of the cells. This work indicates that plating MSCs on soft substrates causes several phenotypic changes which take them a step closer to becoming pluripotent cells. This seems to boost the reprogramming protocol; enhancing the kinetics, robustness of colony formation and reprogramming efficiency. The experimental data fits a theoretical model that correlates low traction force exerted by MSCs with a higher probability of fully reprogramming into iPSCs. We present some evidences that this may be accomplished through the modulation of chromatin structure (Fig. 6A). Indeed, several strategies have been used, in the last years, to generate iPSCs that follow the same idea that reprogramming chromatin is essential to alter cell state. Most studies have used biochemical approaches to do that based on chromatin modulating drugs that change the activity of intrinsic factors, like enzymes^65–67^. We believe that these approaches need to be complemented through the use of extrinsic cues that are sensed from the extracellular environment and boost the intrinsic machinery. In a recent study (Lima *et al*.)^44^, using the same viral vector and similar transduction protocol as described here, we showed that transiently changing a biophysical parameter such as the osmotic pressure of the extracellular media impacts on chromatin structure, transcription and reprogramming kinetics. Both CD34^+^ mononuclear hematopoietic stem cells and fibroblasts experienced an increase in euchromatic content and reprogramming kinetics when subjected to transient hypoosmotic stimuli. Here we show that changing the biophysical parameter stiffness of the extracellular environment translates into a different activity of the intrinsic molecular players and reprogramming kinetics. These complementary approaches follow the same common idea that cell state is influenced by the way cells sense their environment.

Moreover, we show that bona fide stable iPSC colonies plated on soft versus stiff substrates acquire a 3D-like topography, formed by flat cells only in the periphery and more columnar-shaped cells in the center of the colony. This soft phenotype resembles an epithelial-like morphology, supported by the observation of organized vinculin at apical cell-cell contacts. Further studies are needed to address the impact of these different mechanosignaling patterns in pluripotent stem cell potential.

## Materials and Methods

### Preparation of substrates for cell culture

All the substrates used for cell culture, including polydimethylsiloxane (PDMS – 1.5 and 15 kPa plates from Ibidi), tissue culture polystyrene plates (TCPs – Corning-Costar), and glass coverslips (VWR) were functionalized with 15 µg/ml fibronectin (FN – Merck Millipore) before cell seeding. To coat TCPs, the bottom of the culture dishes was covered with the FN solution and incubated for 2 h at 37 °C. For PDMS and glass coverslips, the functionalization with FN was preceded by a specific treatment to reduce its hydrophobicity. First, these platforms were incubated with a solution of dd water/hydrogen peroxide /hydrochloric acid in a volumetric proportion of 5:1:1 (for PDMS) or 1 M NaOH (for glass coverslips) to create silanol groups on the surface, turning the substrates more hydrophilic, and then by addition of 3-APTMS (3-aminopropyltrimethoxisilane)^68,69^ and glutaraldehyde^70,71^ to allow the covalent binding of FN to the PDMS/glass coverslips surface. For these two platforms, the FN solution was incubated for 4 h at 37 °C.

### Cell culture

All cell types used were maintained at 37 °C in humidified incubators with 5% CO_2_/95% air in the specific culture media described below.

Human umbilical cord mesenchymal stem/stromal cells (MSCs) were previously isolated and expanded until passage 2 or 3 and cryopreserved in liquid nitrogen. All samples used in the current study have been thoroughly characterized in our previous report^72^, fulfilling the ISCT criteria that define MSCs^73^, namely being plastic adherent, presenting extensive proliferative capacity in standard conditions, possessing clonogenic capacity (CFU-F assays), expressing and lacking the expression of the expected positive and negative markers of MSCs (CD73, CD90 & CD105, and CD11b, CD34, CD45 & NGFR, respectively) and being able to differentiate into adipogenic, chondrogenic and osteogenic lineages. For the current study, cells were thawed and cultured in Alpha-MEM (minimal essential medium) — Life Technologies — containing 10% (v/v) MSC-qualified fetal bovine serum (Hyclone, GE Healthcare), 100 U/mL Penicillin, 100 μg/mL Streptomycin and 2.5 μg/mL Amphotericin B (all from Life Technologies), as detailed previously^72^. For all experiments, MSCs were used between passages 3 and 4. Human umbilical cords were obtained after birth from healthy donors upon informed consent from the parent(s) and the study was approved by the Ethics Committee of Maternidade de Bissaya Barreto – Centro Hospitalar de Coimbra (ref. 356/Sec). All methods were carried out in accordance with national and European guidelines and regulations.

The human embryonic kidney cell line (293 T – ATCC CRL-3216) was cultured in RPMI medium (Life Technologies) supplemented with 10% FBS (v/v), 100 U/mL penicillin and 100 µg/mL streptomycin.

The reprogrammed cells (iPSCs) were cultured on top of a feeder cell layer. The feeder layer was prepared from mouse embryonic fibroblasts (MEFs – GlobalStem GSC-6001). Before inactivation, MEFs were expanded in DMEM (Life Technologies) supplemented with 10% FBS (v/v), 100 U/mL penicillin and 100 µg/mL streptomycin. After 2 passages, the MEFs were inactivated with 8 μg/mL mitomycin C for 2 hours at 37 °C.

iPSCs were cultured in mTeSR medium (Stemcell Technologies), incubated with 10 µM of ROCK inhibitor Y-27632 (Tocris) for 1 h before and ON after being split.

### Reprogramming of human mesenchymal stem/stromal cells (MSCs)

For MSCs reprogramming, conditioned hESCs/hiPSCs medium was used. The medium is a standard hESCs/hiPSCs formulation^74^ [composed by KO-DMEM, 20% (v/v) KO Serum replacement, 1% (v/v) nonessential amino acids (all from Life Technologies), 1 mM L-Glutamine, 0.1 mM β-mercaptoethanol (Sigma), 4 ng/ml basic Fibroblast Growth Factor (bFGF, Peprotech) and 100 U/mL of penicillin and 100 µg/mL of streptomycin] that was conditioned by incubation with inactivated MEFs for 24 h.

For reprogramming, after transduction (detailed below), MSCs were harvested and seeded in parallel onto 1.5 and 15 kPa PDMS plates and TCPs at a density of 500 cells/cm^2^, and maintained in culture for 7 days with hESCs/hiPSCs conditioned medium, with daily medium change.

After 7 days in culture, newly formed iPSC colonies were manually picked, seeded and expanded in a feeder system with inactivated MEFs on a regular (GPa range stiffness) tissue culture polystyrene 6-well plate (Corning-Costar), for 6/7 passages. After the manual picking expansion, the colonies were dissociated using 1 mg/ml Collagenase IV (Life Technologies) and further expanded.

### Lentiviral production

The viral packaging was performed in 293 T cells with the appropriate amounts of transfection agent Lipofectamine 2000 (Thermo-Fisher) and the plasmids of interest. The lipofectamine was mixed with DMEM without serum and incubated at room temperature for 10 minutes. After incubation, the plasmids were added and maintained at room temperature for 30 minutes. Lastly, this mixture was added to 70%-80% confluent 293 T culture flasks (T75 flask). The 293 T medium was changed by fresh medium after 16 h of incubation (at 37 °C).

Three days after transfection, the 293 T viral supernatants were collected into 50 mL tubes and spun at 300 *g* for 5 minutes to pellet the cellular debris. After centrifugation, the supernatants were filtered (0.4μm syringe filter) into centrifuge tubes and centrifuged at 19,000 *g* for 4 hours. The lentiviral particles were then resuspended in 200 μL of PBS and stored in aliquots at −80 °C.

### Lentiviral transduction of MSCs with a polycistronic reprogramming vector

A lentiviral polycistronic self-inactivating vector expressing the four Yamanaka factors was used to perform MSCs reprogramming^49^. Besides from encoding the human reprogramming factors (Oct4, Klf4, Sox2, c-Myc) this lentiviral vector has a fluorescent reporter (dTomato), which allows monitoring of the success of infection and evaluate the expression of the reprogramming factors within the transduced cells^49^. Shortly after infection, several MSCs strongly display the fluorescent reporter (dTomato fluorescent signal), indicating that the reprogramming factors within the vector are being expressed. This vector has been shown to be rapidly silenced upon epigenetic modifications intrinsic to the cellular reprogramming process. Therefore, the endogenous expression of pluripotency-related genes is activated in successfully reprogrammed cells, where the vector becomes silenced by losing expression of the exogenous reprogramming factors and the fluorescent reporter^49^. Therefore, the fully reprogrammed cells/colonies no longer express the fluorescent reporter but present the morphological features of pluripotent colonies and express pluripotency-related markers (Fig. S2, Fig. S4). For lentiviral transduction, the lentiviral particles (20 µl) were added onto a 6-well plate (in DMEM) previously coated with 15 µg/cm^2^ retronectin (Takara) and centrifuged for 1 h, 2,000 *g* at 32 °C. After centrifugation, the supernatant was removed, and the cell suspension of MSCs was added immediately (16,000 cells/cm^2^) to the plate and incubated for 12 h at 37 °C and 5% CO_2_. After 12 h, an additional load of lentiviral particles (10 µl) was added directly to the cells in culture and incubated for another 12 h in the same conditions, and this step was repeated once more. Next, the medium was refreshed, and the cells were further incubated for 24 h. Then, the transduced cells were re-seeded (500 cells/cm^2^) onto the distinct substrates (1.5 and 15 kPa PDMS or TCPs) and maintained for 7 days in culture during the reprogramming process.

### Immunocytochemistry, microscopy and image analyses

For immunostaining, cells were fixed with 4% (w/v) paraformaldehyde (Sigma), permeabilized with 0.1% Triton X-100 (Sigma) and blocked with 1% (w/v) BSA (Calbiochem) in PBS. For actin-cytoskeleton staining, samples were incubated with TRITC-labeled Phalloidin (Life Technologies) for 1 hour at room temperature. The incubation with the primary antibodies [anti-TRA-1–60, anti-TRA-1–81, anti-SSEA4 (all from Cell Signaling Technology), mouse anti-vinculin (Abcam) and rabbit anti-connexin 43 (Sigma)] was done for 2 hours at room temperature, followed by three washing steps with PBS and incubation with Alexa 488- /Alexa 568- / Alexa 647-labelled secondary antibodies (Molecular Probes – Life Technologies) for 1 h at room temperature. Nuclei were stained with 0.8 µg/mL DAPI (Molecular Probes – Life Technologies) for 5 minutes (for fixed cells) or with 1 µg/ml Hoechst 33342 (Sigma Aldrich) added to the culture media for 10 min (for live cells).

Fluorescence microscopy images were acquired using a Zeiss Axiovert 200 M fluorescence microscope and AxioVision release 4.8 software (Zeiss).

DAPI images were used to quantify the mean of fluorescence intensity (MFI) and the area of cellular nuclei. For MFI quantification of DAPI and H4K16ac signal, regions of interest (ROIs) were defined based on the DAPI signal threshold that was adjusted until all nuclei were selected. To analyze the area of focal adhesions (FAs), ROIs were defined by adjusting the anti-vinculin signal threshold within the lamellar zones of cells (represented by white rectangles – Fig. 2A). For MFI quantification of F-actin, ROIs were defined delimiting cells by the edges. Image processing and analysis were performed with the FIJI software.

Confocal microscopy was performed using a Zeiss LSM 710. Z-stack image series were acquired using objective optimal pinholes and steps were matched to the different pinholes for optimal coverage of the entire volume of the colonies and cells. Orthogonal projections were used to measure shape descriptors including aspect ratio features in ImageJ. iPSC Colony characterization was done in Cell Profiler using Hoechst 33342 staining to segment nuclei and obtain measures of object size, shape and intensity.

### Nuclear extracts and western blotting analysis

To obtain nuclear protein extracts from MSCs, 24 h after culture on substrates with distinct stiffness, cells were washed with PBS and then scraped in Laemmli buffer. The subcellular fractionation was done following the protocol described in^75^, and nuclear fractions were collected into microtubes and then heated at 95 °C for 5 min. Next, the samples were spun down and passed ten times through a 25 G needle and further analyzed by Western blot. Proteins were separated by SDS-PAGE [12.5% (w/v) acrylamide–bisacrylamide (Bio-Rad) gels] and transferred onto PVDF membranes that were subsequently probed with specific antibodies against H4K16ac (Abcam) and H4 (Cell Signaling) followed by the incubation with the respective alkaline phosphatase-conjugated secondary antibodies (Jackson ImmunoResearch). The membranes were incubated for 5 minutes with enhanced chemifluorescence substrates (ECF – GE Healthcare) and imaged in a Molecular Imager FX Pro Plus system (BioRad) using the Quantity One software (BioRad). The acquired images were analyzed with Image Lab software, version 5.1 (BioRad).

### Real-time quantitative PCR (qRT-PCR) analysis

MSCs were sampled 4 days after seeding on distinct substrates for RNA isolation. RNA was extracted from MSCs using the RNeasy kit (Qiagen) and cDNA was generated by using the MultiScribe™ Reverse Transcriptase kit (Life Technologies), according to the manufacturer’s instructions. Gene expression profile for specific targets was evaluated by qRT-PCR. The analysis was performed using primers purchased from Sigma corresponding to *Homo sapiens* sequences. The primers were as follows: *Beta-2 microglobulin* forward, 5′-GGGTTTCATCCATCCGACATTG-3′, reverse, 5′-TGGTTCACACGGCAGGCATAC-3′; *Nanog* forward, 5′-TCTCCAACATCCTGAACCT-3′, reverse, 5′-GCGTCACACCATTGCTAT-3′; *Sox2* forward, 5′-GATGGTTGTCTATTAACTTGTTCA-3′, reverse, 5′-TCTCTCCCTTTCTTTCTCTCT-3′; *Oct-4* forward, 5′-GTGGAGGAAGCTGACAACAA-3′, reverse, 5′-CTCCAGGTTGCCTCTCACTC-3′. qRT-PCR was performed on an ABI PRISM 7500 System (Applied Biosystems) using Power SYBR Green PCR Master Mix (Life Technologies). Data analysis was done using the ∆∆C_T_ method.

### Mathematical model

The reprogramming rate of a cell is given by

2 $$K=\frac{dR}{dt}={K}_{0}+{K}_{1}F$$

where *K*_0_ and *K*_1_ are two parameters adjusted to data, given in Supplementary Table S2, and *F* is the total traction force of the cell on the substrate. The reprogramming starts at 0 and its rate of change with time is a function of *F*, and then of *E* and the area of contact between cell and substrate.

The parameters *K*_0_ and *K*_1_ are adjusted to the experimental results obtained in this work, as the reprogramming time in a soft substrate (*E* = 1.5 kPa) is about 5 days and in a rigid substrate (*E* = 1 GPa) is about 7 days. The reprogramming time *t*_*R*_ is given by, from equation 2,

3 $${t}_{R}=\frac{{R}_{th}}{K}$$

with *R*_*th*_ being the threshold of the reprogramming level when the fluorescent reporter protein (dTomato) is silenced.

A simulation run is started with a single PSC at the center of the domain, with a reprogramming level *R*=0 and traction force *µ*, in a substrate with Young’s modulus *E* (as given by Equation 1). When culture time advances, in units of Monte Carlo Steps (MCS), the cell proliferates, with an average cell cycle of 16 hours, forming a colony. As shown before the reprogramming level increases with time at a rate given by Equation 2; when it passes the threshold, *R*=1, the cell is considered fully reprogrammed as an iPSC.

In the CPM^3^ the cells’ energy has two terms, one on their adhesion and the other on their area (the simulation is done in two dimensions). The energy Hamiltonian is then given by

4 $$H={H}_{adhesion}+{H}_{area}=\sum _{ < ij > }{J}_{\tau (\sigma (i))\tau ^{\prime} (\sigma (j))}(1-{\delta }_{\sigma (i)\sigma (j)})+\sum _{\sigma =1}^{N}{\lambda }_{\tau (\sigma )}{(\frac{A(\sigma )-{A}_{\tau (\sigma )}^{T}}{{A}_{\tau (\sigma )}^{T}})}^{2}$$

where *J*_*ττ*′_ is the adhesion energy cost between cells of type *τ* and *τ’*, a sum over < *ij* > means a sum over element *i* and over every neighbor *j* of *i*; *λ*_*τ*_ describes the energy cost of a deviation from the cell target area *A*_*τ*_^*T*^. A Finite Element Method (FEM) coupled to this model calculates the strain and stress on the ECM. This model has been used in the past to simulate the vascular formations created by endothelial cells in matrices of different rigidities. This phenomenon hinges on the ability of endothelial cells using strain to polarize themselves. Stem cells, on the other hand, tend to be circular, and to not have this polarization capacity. Therefore, the term that describes the dependence of the energy on the local strain was omitted from equation (4).

This is a stochastic model and the results change from run to run. The variability is introduced in the parameters *K*_0_ and *K*_1_ of each individual cell in the simulation. The reprogramming time is considered to be Gaussian distributed around an average value, with *σ* = 26% of the mean.

### Statistical analysis

Statistical analyses were performed with GraphPad Prism 7.0 software and the tests applied to each dataset are detailed in the corresponding figure legend. Values represent the mean ± SEM of at least 3 independent experiments (*P < 0.05; **P < 0.01; ***P < 0.001 and ****P < 0.0001 for statistically significant differences).

## Supplementary information

Supplementary Information

Supplementary Video 1

Supplementary Video 2

## Data Availability

The datasets generated during and/or analysed during the current study are available from the corresponding authors on reasonable request.
