# Supplementary material for: Soft culture substrates favor stem-like cellular phenotype and facilitate reprogramming of human mesenchymal stem/stromal cells (hMSCs) through mechanotransduction
Source: Sci Rep. 2019 Jun 24;9:9086. doi: 10.1038/s41598-019-45352-3 (PMC6591285; doi:10.1038/s41598-019-45352-3)
Supplement: Supplementary file 1 — Supplementary Information [file 41598_2019_45352_MOESM1_ESM.pdf]

Title

Soft culture substrates favor stem-like cellular phenotype and facilitate reprogramming of human mesenchymal stem/stromal cells (hMSCs) through mechanotransduction

Authors

Heloísa Gerardo[1], Ana Lima[1,2], João Carvalho[3], João R. D. Ramos[4], Sofia Couceiro[5], Rui D. M. Travasso[3], Ricardo Pires das Neves[1,6,*] & Mário Grãos[1,6,7,*]

Affiliations

[1] CNC — Center for Neuroscience and Cell Biology, University of Coimbra, UC-Biotech Building, Biocant Park, Cantanhede, Portugal

[2] Faculty of Science and Technology, University Nova of Lisbon (MIT-Portugal PhD Program), Caparica, Portugal

[3] Centro de Física da Universidade de Coimbra (CFisUC), Department of Physics, University of Coimbra, Coimbra, Portugal

[4] Max Planck Institute for Dynamics and Self-Organization, Göttingen, Germany

[5] Stemlab S.A. (Crioestaminal), Biocant Park, Cantanhede, Portugal

[6] Institute for Interdisciplinary Research, University of Coimbra, Coimbra, Portugal

[7] Biocant, Technology Transfer Association, Cantanhede, Portugal

[*] R.P.N. and M.G. are both senior and corresponding authors of this study.

Supplementary Figure Legends

Figure S1. Substrate rigidity as a modulation tool of chromatin status. Representative images of MSCs cultured on stiff, 15kPa and 1.5kPa PDMS substrates, showing different levels of chromatin condensation based on **(A)** DAPI and **(B)** H4K16ac staining. **(A)** Higher levels of MFI in DAPI-stained nuclei (pseudo colored for better visualization — lower panel in A) correspond to compacted chromatin. The nuclei of cells plated on stiff substrates (TCPs) are typically more heterochromatic than on soft substrates (15 or 1.5 kPa PDMS). **(B)** Conversely, by analyzing the labelling of H4K16ac, a histone modification that co-localizes with permissive euchromatic regions where transcription occurs, higher levels of MFI (pseudo color—lower panel in B) correspond to euchromatic regions and can be readily observed in nuclei of cells cultured on soft substrates (1.5 or 15 kPa PDMS) when compared with the stiff substrate (glass coverslips).

Figure S2. Colony formation profile during MSCs reprogramming using a lentiviral polycistronic all-in-one self-inactivating vector. After the transduction protocol using a lentiviral reprogramming vector (Fig. 4A), MSCs were re-plated on distinct substrates and then cultured for 7 days. Newly formed hiPSC colonies could be observed on all tested substrates. The polycistronic viral vector encodes for the fluorescent reporter protein (dTomato), which is expressed in transduced cells, but becomes epigenetically silenced in fully-reprogrammed cells (those which began to express their endogenous pluripotency genes). Both panels show colonies with typical iPSC morphology, but in **(A)** colonies are partially reprogrammed, still expressing the exogenous pluripotency factors as evidenced by the presence of the fluorescent reporter protein dTomato, while in **(B)** the colony is fully reprogrammed as no signal for dTomato can be detected. Scale bars represent 100 µm.

Figure S3. Frequency of colony formation on distinct substrates. Number of colonies formed per each thousand cells plated. Bars represent mean ± SEM of at least 3 independent

experiments. Statistical analysis was performed using Kruskal-Wallis test followed by Dunn's multiple comparison test.

Figure S4. Reprogrammed cells present hiPSCs morphology and surface markers. The reprogrammed hiPSC colonies obtained maintained the typical morphology during the expansion process for several passages on inactivated MEF feeders, as assessed by **(A)** phase-contrast microscopy and **(B)** expression of pluripotency markers (immunostaining and fluorescence microscopy) TRA-1-81, TRA-1-60 (both in green) and SSEA-4 (red), in contrast to MSCs, which did not express such markers. Scale bars correspond to 100 µm and nuclear counterstaining in fluorescence microscopy images was performed using DAPI (blue).

Figure S5. Orthogonal projections from a representative confocal microscopy Z-stack image series of DAPI stained hiPSCs nuclei on the edge of a colony cultured on 1.5 kPa (soft) PDMS substrate.

Figure S6. Representative confocal microscopy images of hiPSC colonies stained for the nucleus (DAPI), vinculin and F-actin (phalloidin) cultured on stiff or soft substrates (as indicated). The images are representative of the central region of the colonies, at the basal (lower panel) or apical (upper panel) plane. The differences in vinculin and actin staining are evident when comparing colonies cultured on soft versus stiff substrates.

Figure S7. Representative confocal microscopy images of vinculin, actin and conexin-43 stained hiPSC colonies cultured on soft PDMS substrate. In the upper panel is presented the edge of a colony, and the staining variations from the bottom (lower Z) to the top (higher Z) of the colony. In the lower panel is presented the top of the colony with higher magnification. Scale bars correspond to 10 µm and 5 µm in low and higher magnification images, respectively.

Figure S8. Representative confocal microscopy images from the edge of hiPSC colonies cultured on stiff or soft substrates. The staining with DAPI, vinculin and actin evidence differences in the edge of the colonies, depending on the substrate stiffness. Scale bar corresponds to 10 µm.

Supplementary Video 1. Orthogonal Z-stack projection of a DAPI stained hiPSC colony cultured on stiff or soft substrates.

Supplementary Video 2. Basal to apical z-stacks of hiPSC colonies stained for vinculin, F-actin and conexin-43. Colonies were cultured on stiff or soft substrates for 4 days.

Supplementary Tables

Supplementary Table S1. Cellular Potts Model (CPM) parameters.

**Parameter**	**Value**
Pixel size	2 µm
Monte Carlo Step	20 min
Target Area	A^T = 200 µm^2
Area constraint	λ = 250
Cell-substrate energy cost	J_{i0} = 1.0
Cell-cell energy cost	J_{ij} = 1.0

Supplementary Table S2. Other model parameters.

**Parameter**	**Value**
Maximum traction force for E = 0	a = 8.283 nN
Maximum traction force	A = 127.1 nN
Traction force half maximum	b = 23.46 kPa
Reprogramming threshold	R_{th} = 1
Reprogramming rate parameter	K_0 = 0.2082 day^{-1}
Reprogramming rate parameter	K_1 = -150.53 day^{-1} N^{-1}
Substrate Young's modulus	E = 1.5, 15, 1x10^6 kPa

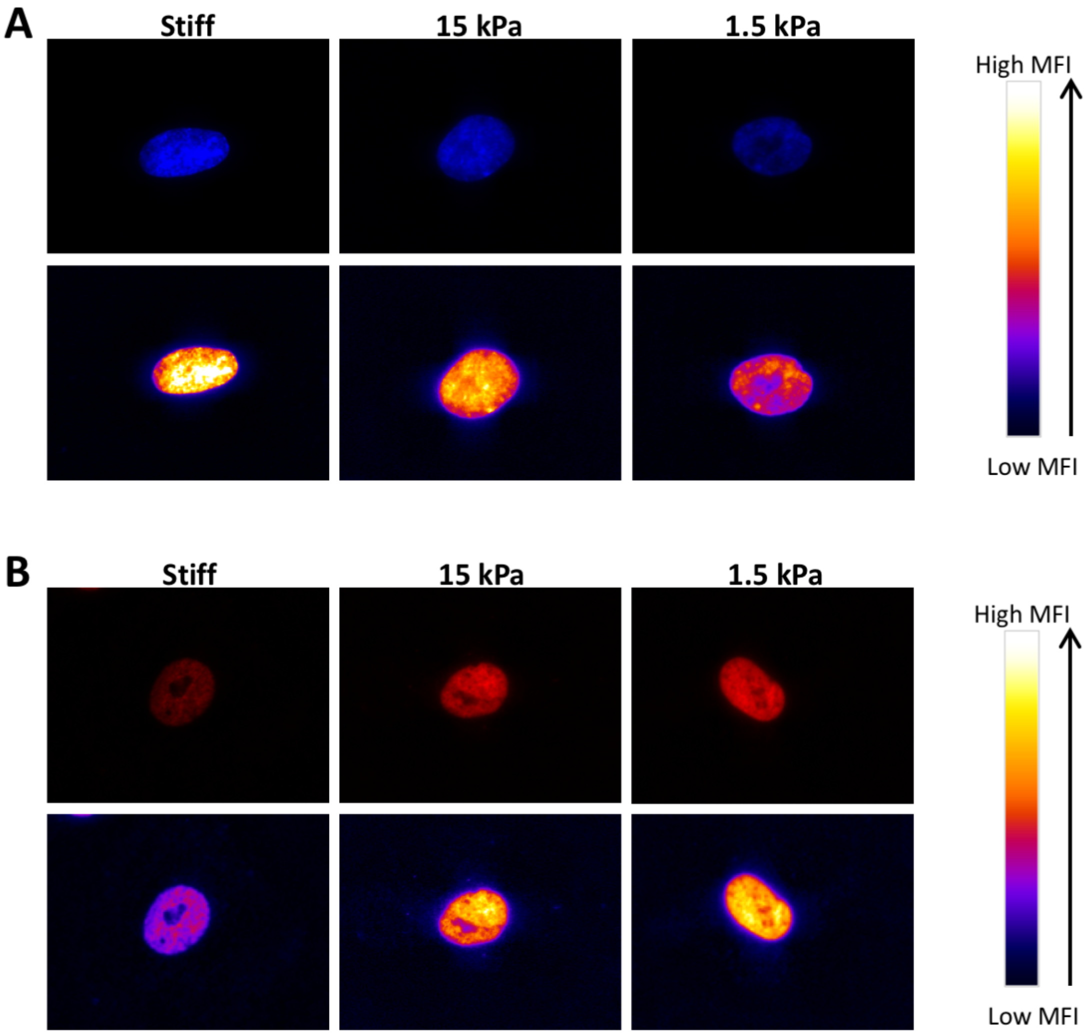
A
Stiff
15 kPa
1.5 kPa
High MFI
Low MFI
B
Stiff
15 kPa
1.5 kPa
High MFI
Low MFI

Supp. Fig S1

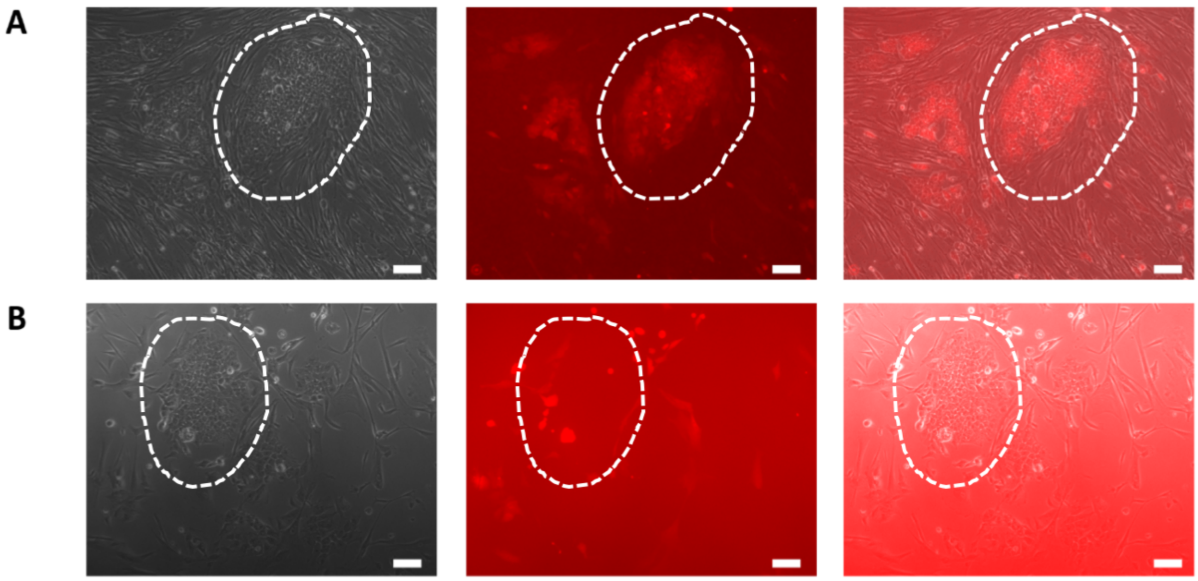

Supp. Fig S2

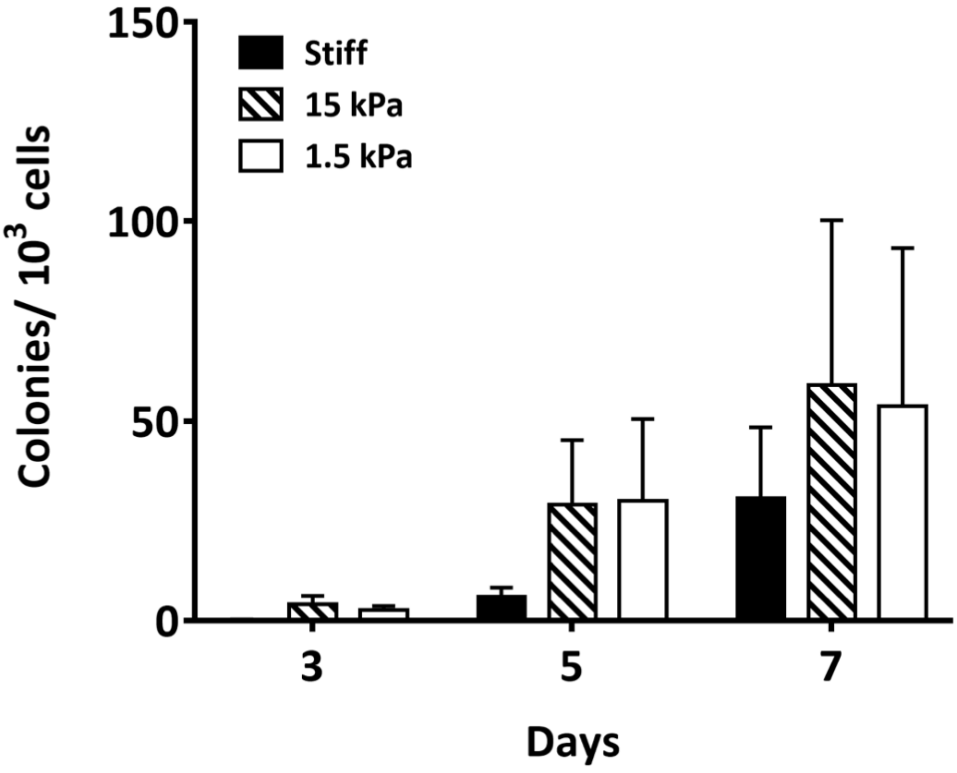

Supp. Fig S3

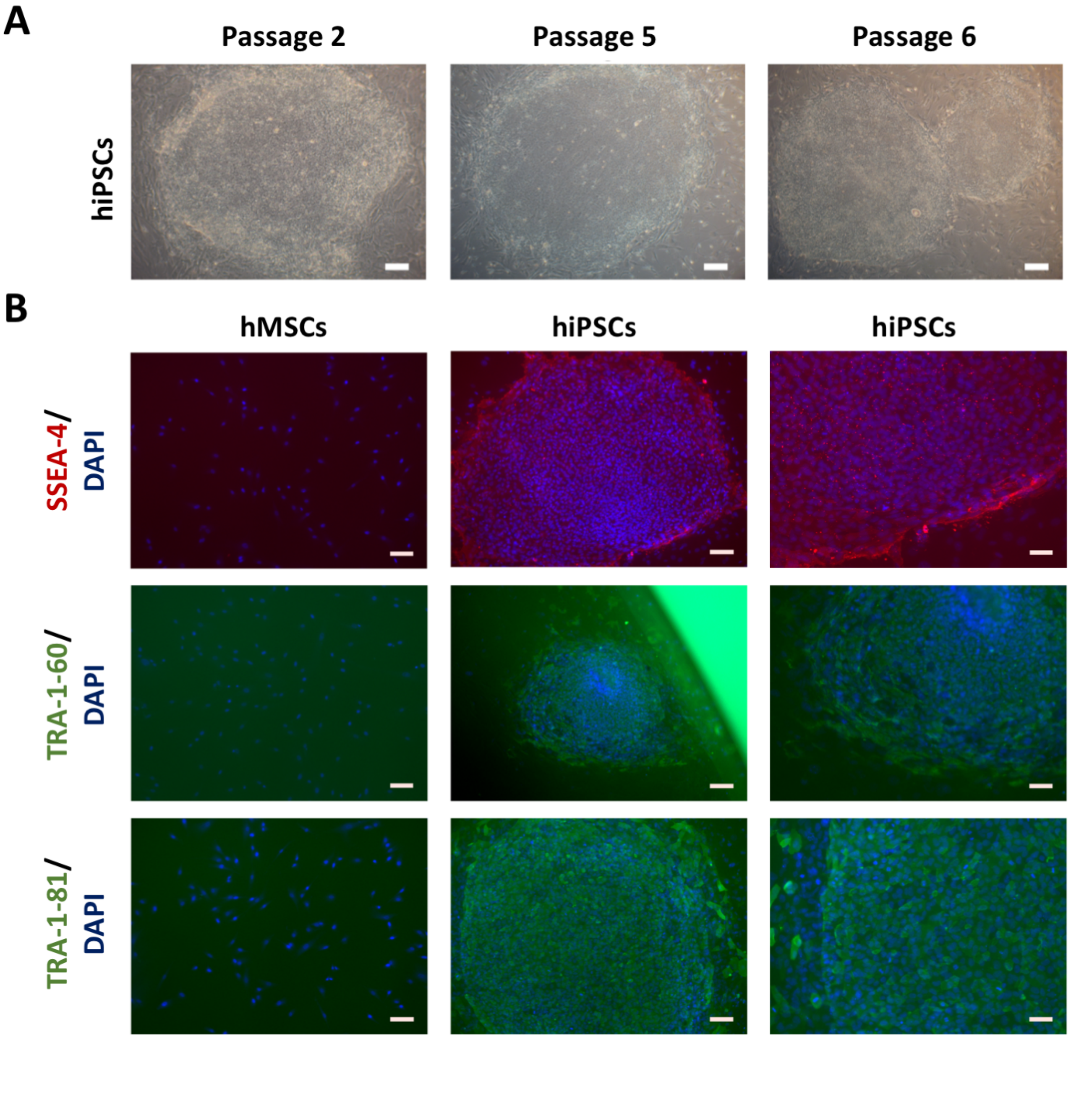
A
Passage 2
Passage 5
Passage 6
hiPSCs
B
hMSCs
hiPSCs
hiPSCs
SSEA-4/
DAPI
TRA-1-60/
DAPI
TRA-1-81/
DAPI

Supp. Fig S4

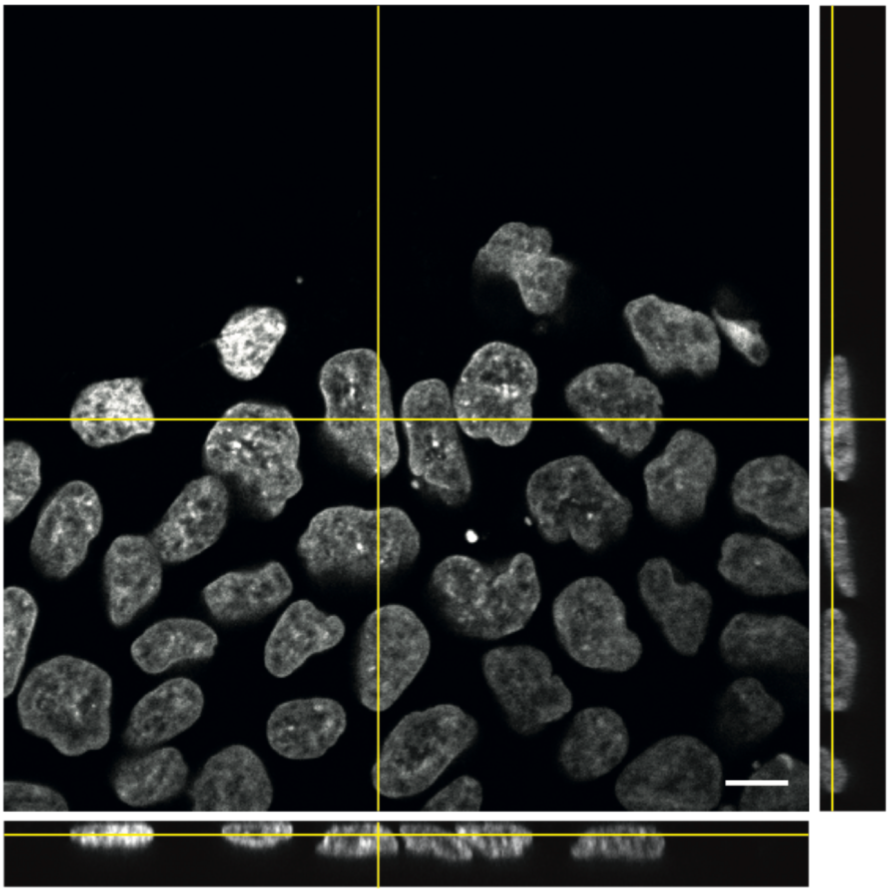

Edge Soft

Supp. Fig S5

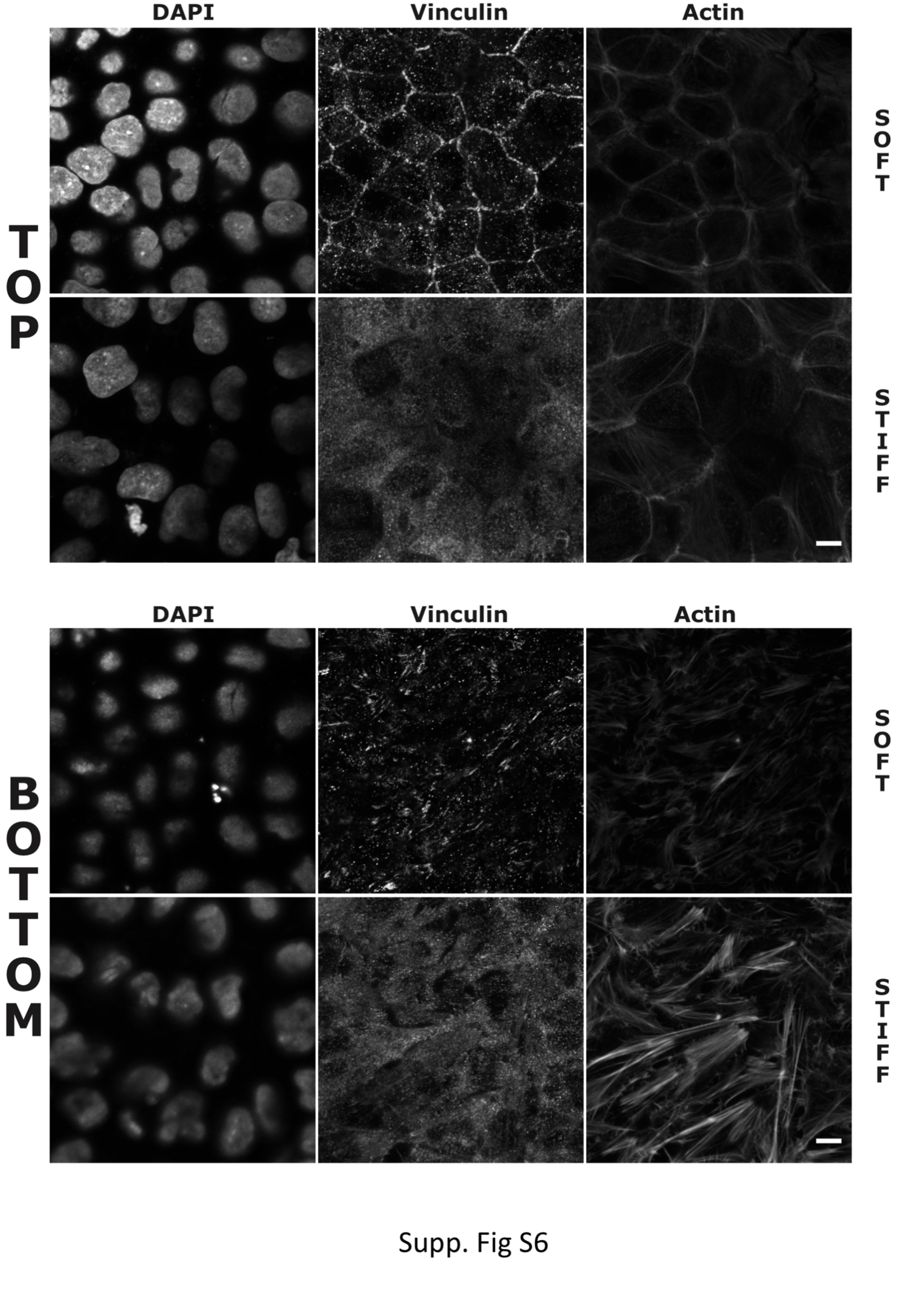
DAPI
Vinculin
Actin
TOP
SOFT
STIFF
DAPI
Vinculin
Actin
BOTTOM
SOFT
STIFF

Supp. Fig S6

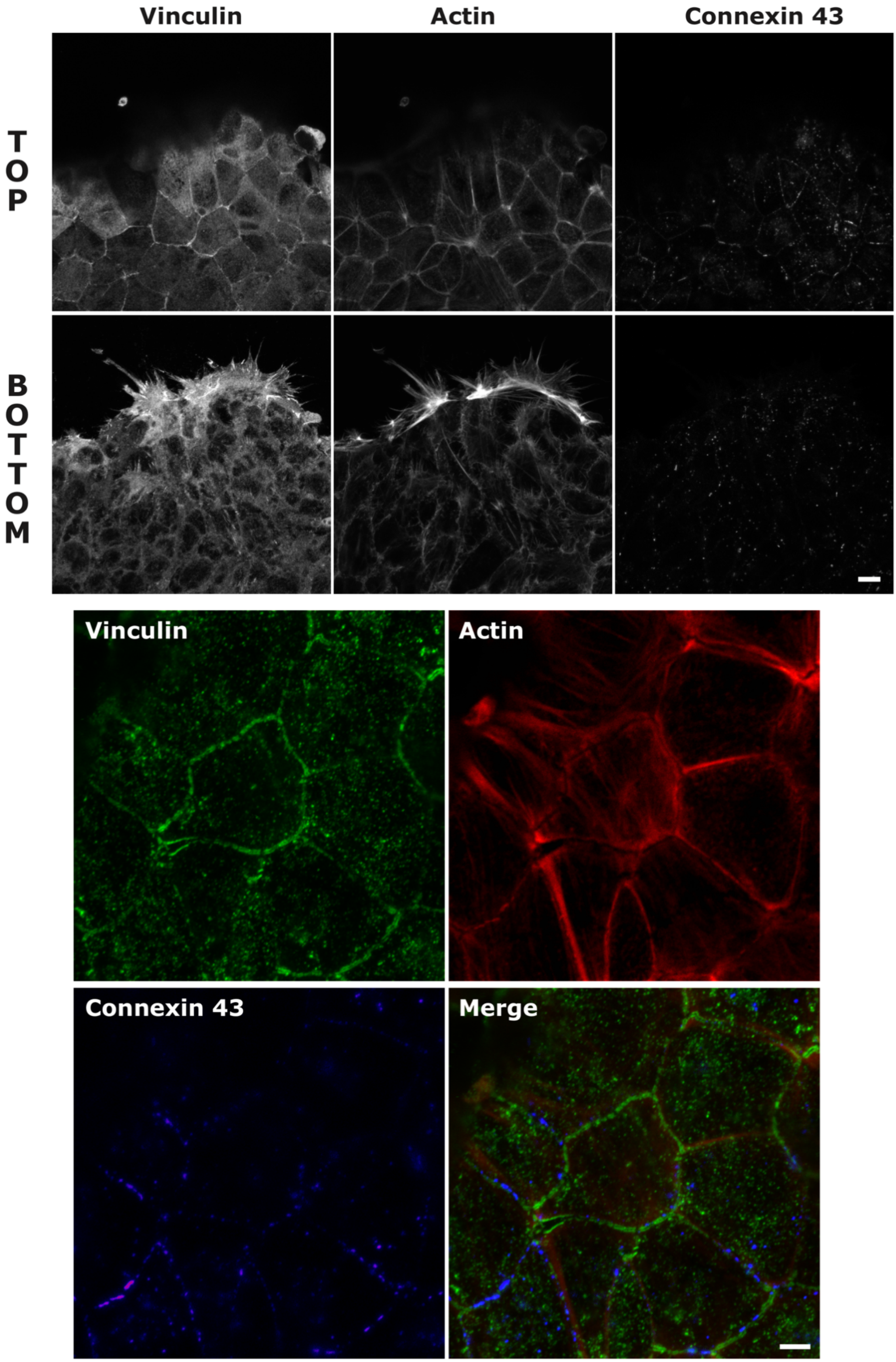
Vinculin
Actin
Connexin 43
TOP
BOTTOM
Vinculin
Actin
Connexin 43
Merge

Supp. Fig S7

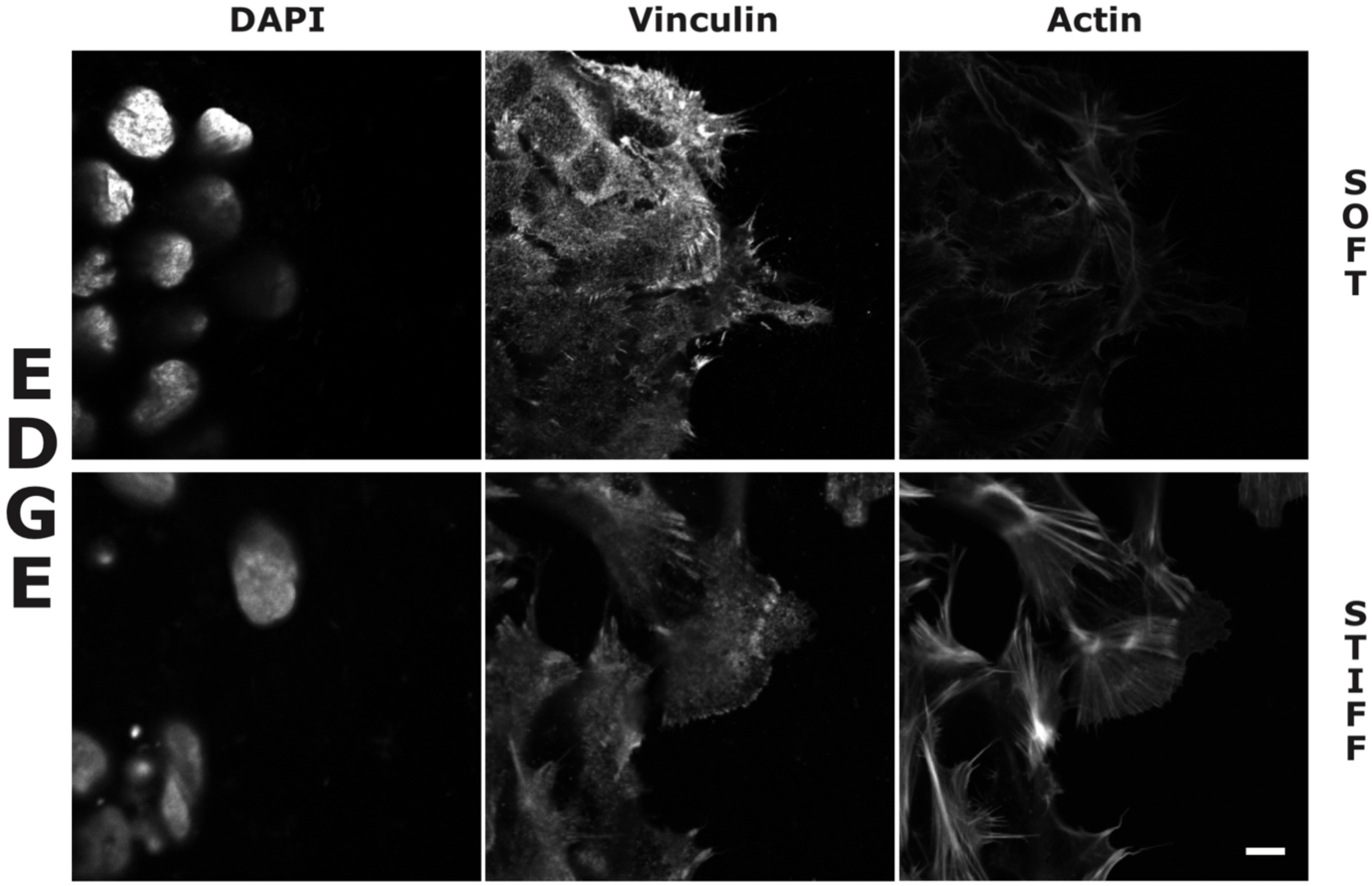

Supp. Fig S8

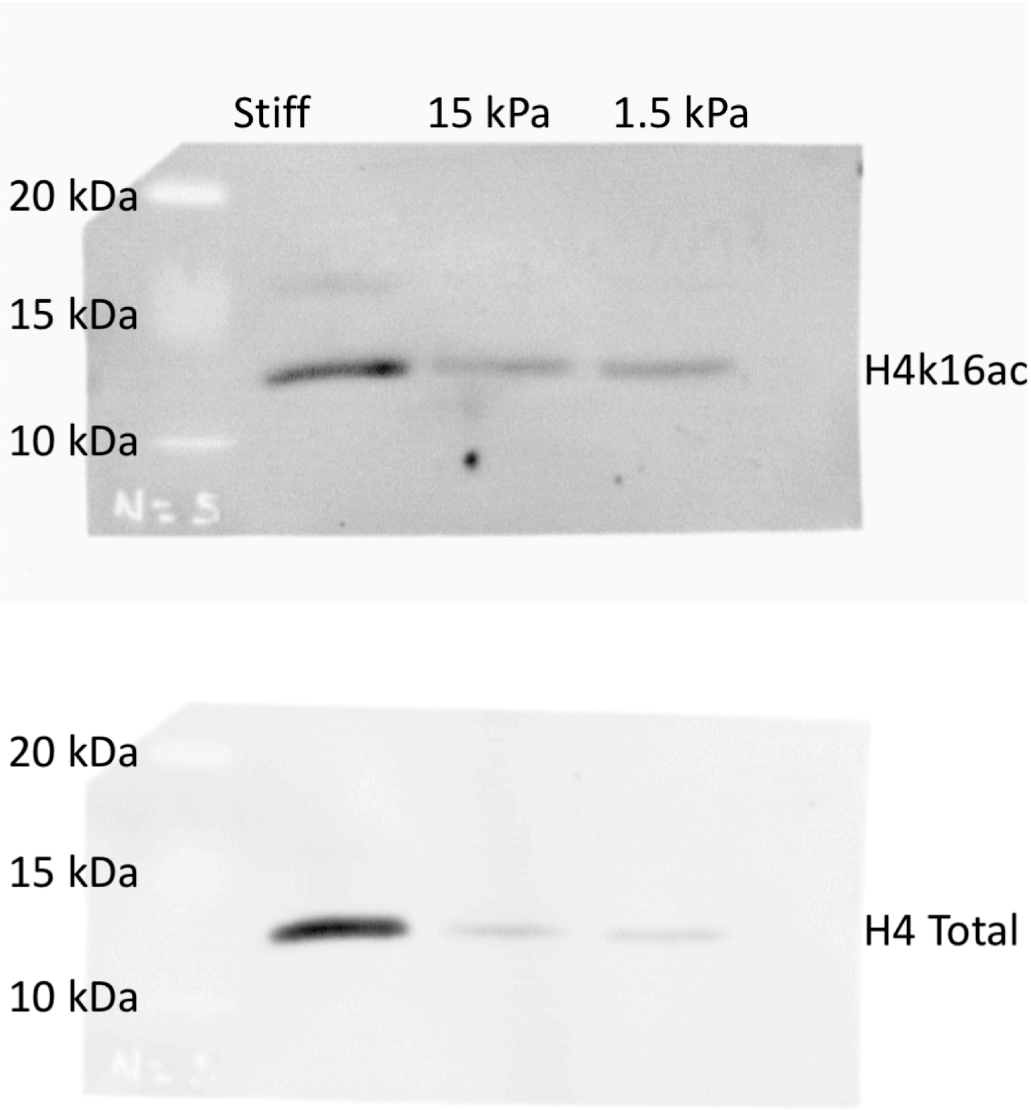

Full length blots used for Figure 3C
